# Supplementary material for: Moving Beyond Simplistic Research Design in Health Professions Education: What a One-Group Pretest-Posttest Design Will Not Prove
Source: MedEdPORTAL. 2025 May 20;21:11527. doi: 10.15766/mep_2374-8265.11527 (PMC12089416; doi:10.15766/mep_2374-8265.11527)
Supplement: Supplementary file 1 — Presentation for Research in HPE.pptxLesson Plan - 60 minutes - In Person.docxLesson Plan - 60 minutes - Virtual.docxLesson Plan - 75 minutes - In Person.docxLesson Plan - 75 minutes - Virtual.docxCase Study and Internal Validity Handout.docxEvaluation Form.docx [file mep_2374-8265.11527-s001.zip › A. Presentation for Research in HPE.pptx]

## Slide 1
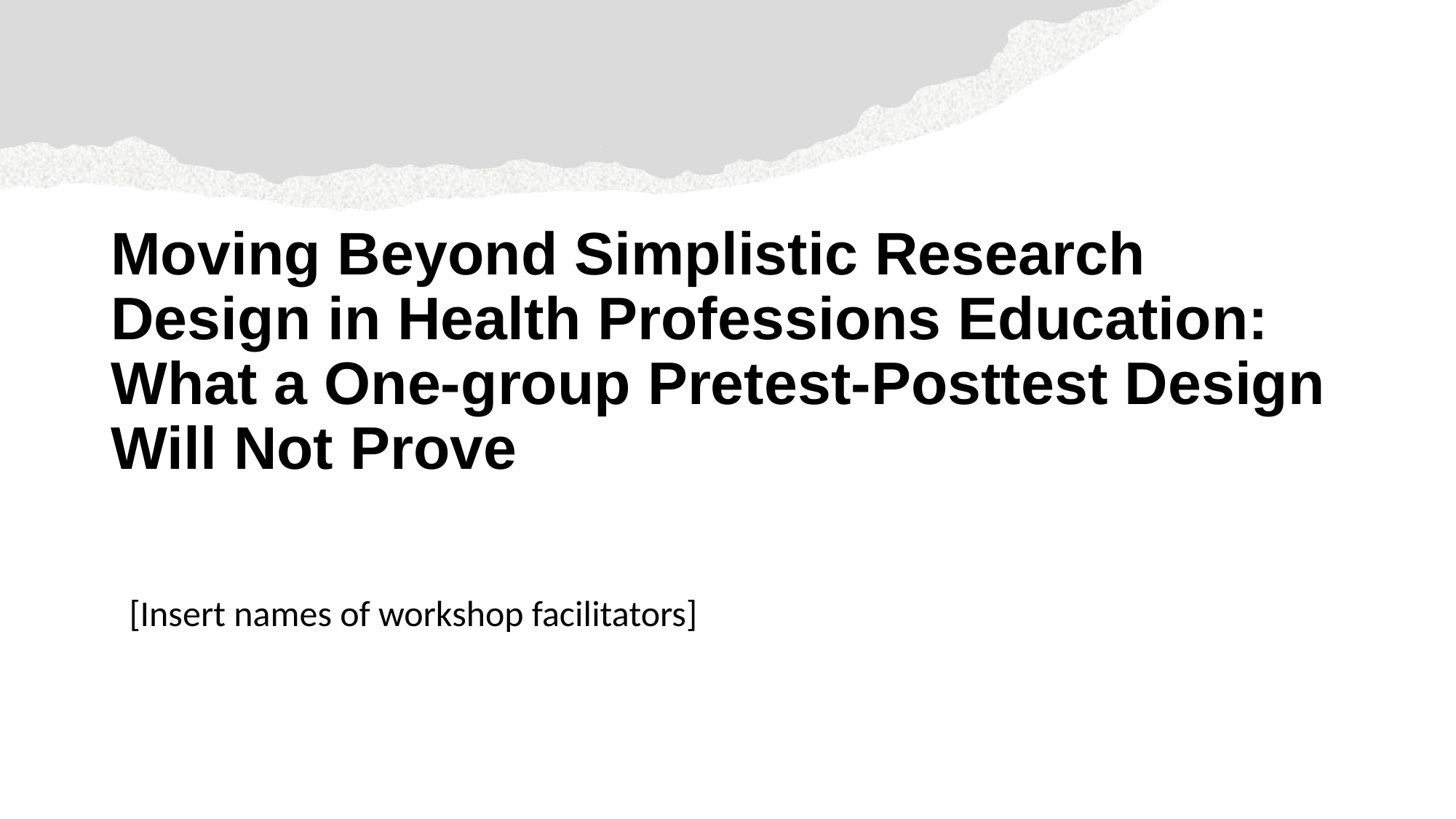

# Moving Beyond Simplistic Research Design in Health Professions Education: What a One-group Pretest-Posttest Design Will Not Prove
[Insert names of workshop facilitators]

## Slide 2
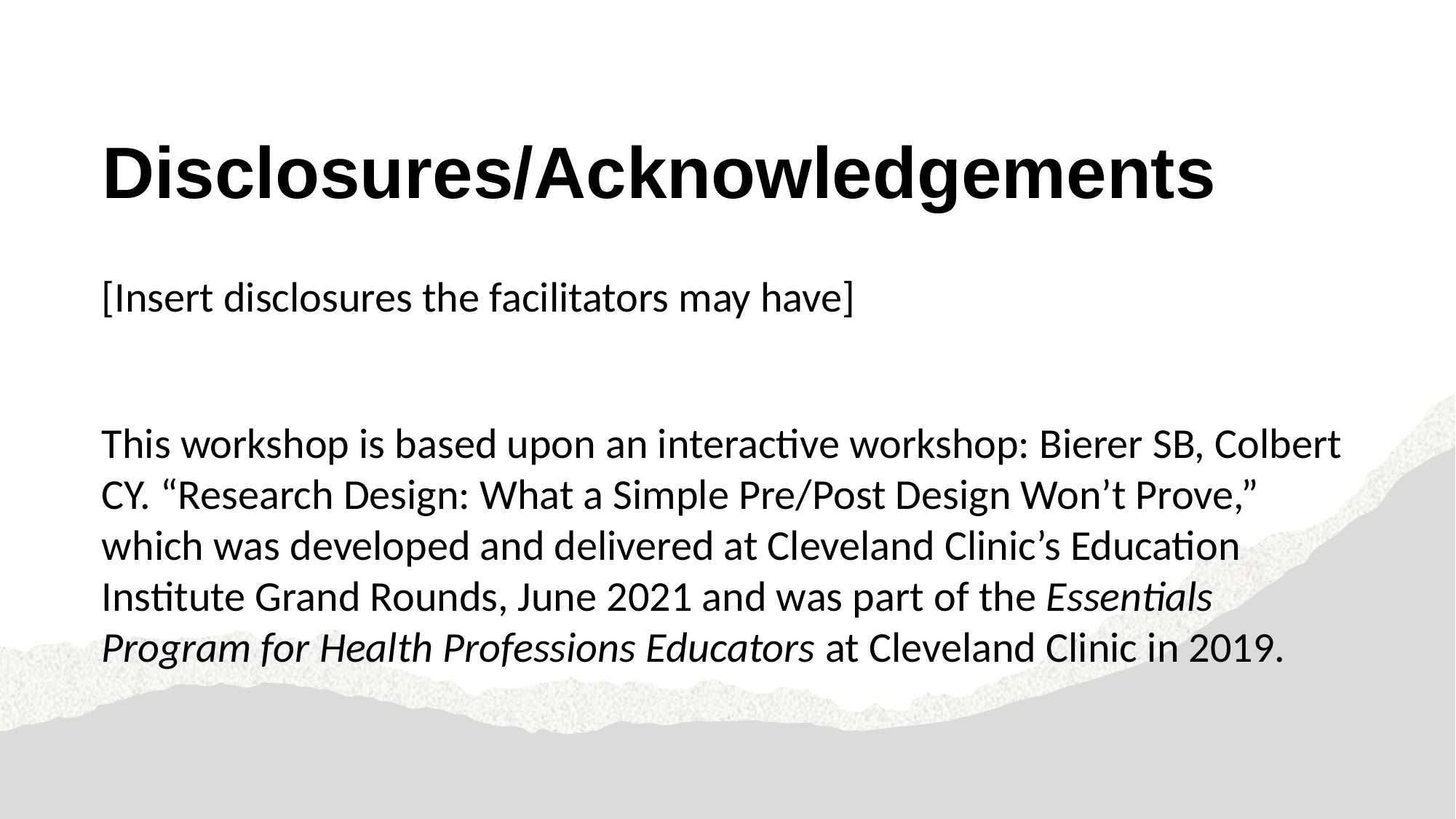

# Disclosures/Acknowledgements
[Insert disclosures the facilitators may have]
This workshop is based upon an interactive workshop: Bierer SB, Colbert CY. “Research Design: What a Simple Pre/Post Design Won’t Prove,” which was developed and delivered at Cleveland Clinic’s Education Institute Grand Rounds, June 2021 and was part of the Essentials Program for Health Professions Educators at Cleveland Clinic in 2019.

## Slide 3
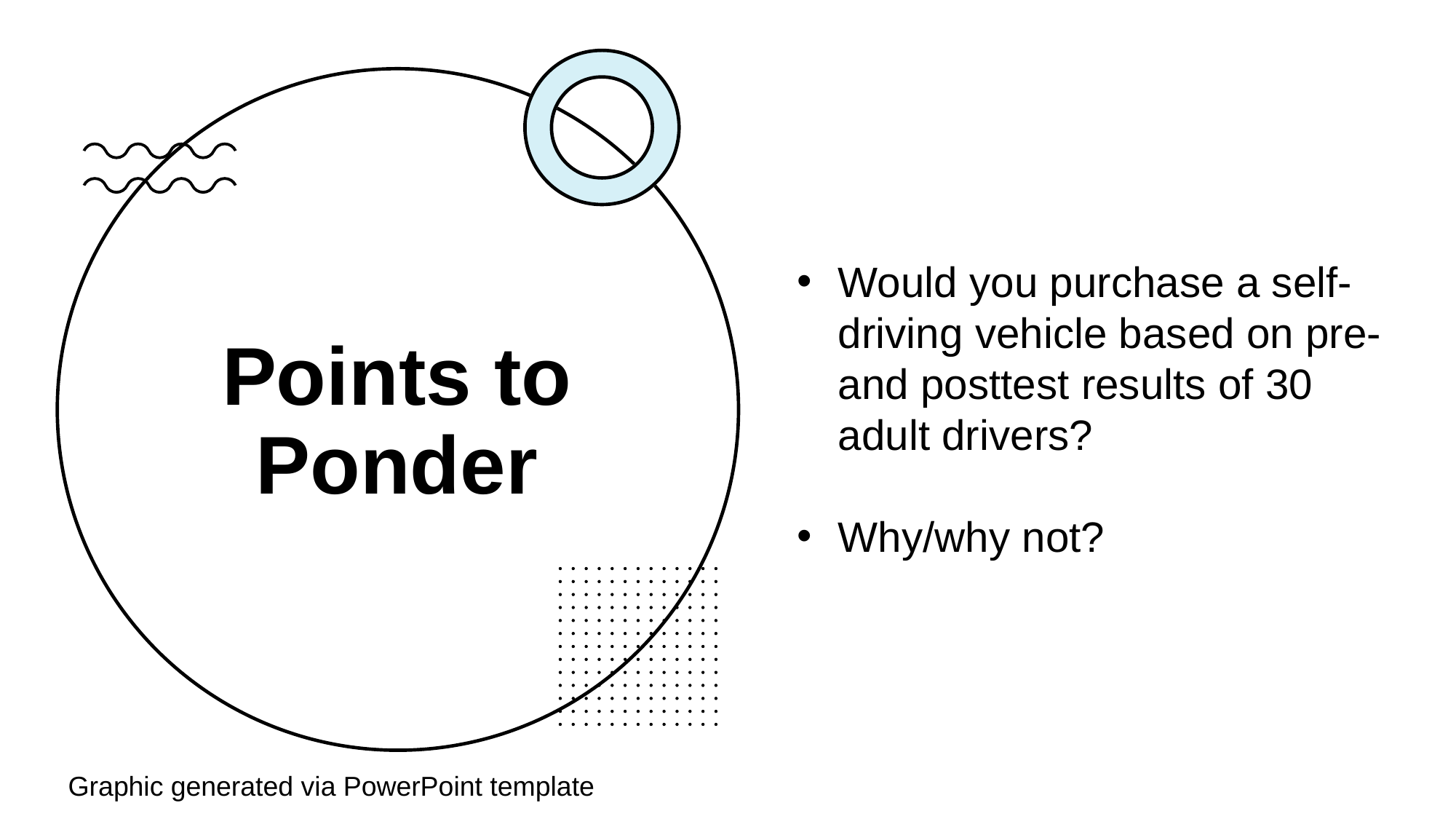

# Points to Ponder
Would you purchase a self-driving vehicle based on pre- and posttest results of 30 adult drivers?
Why/why not?
Graphic generated via PowerPoint template

## Slide 4
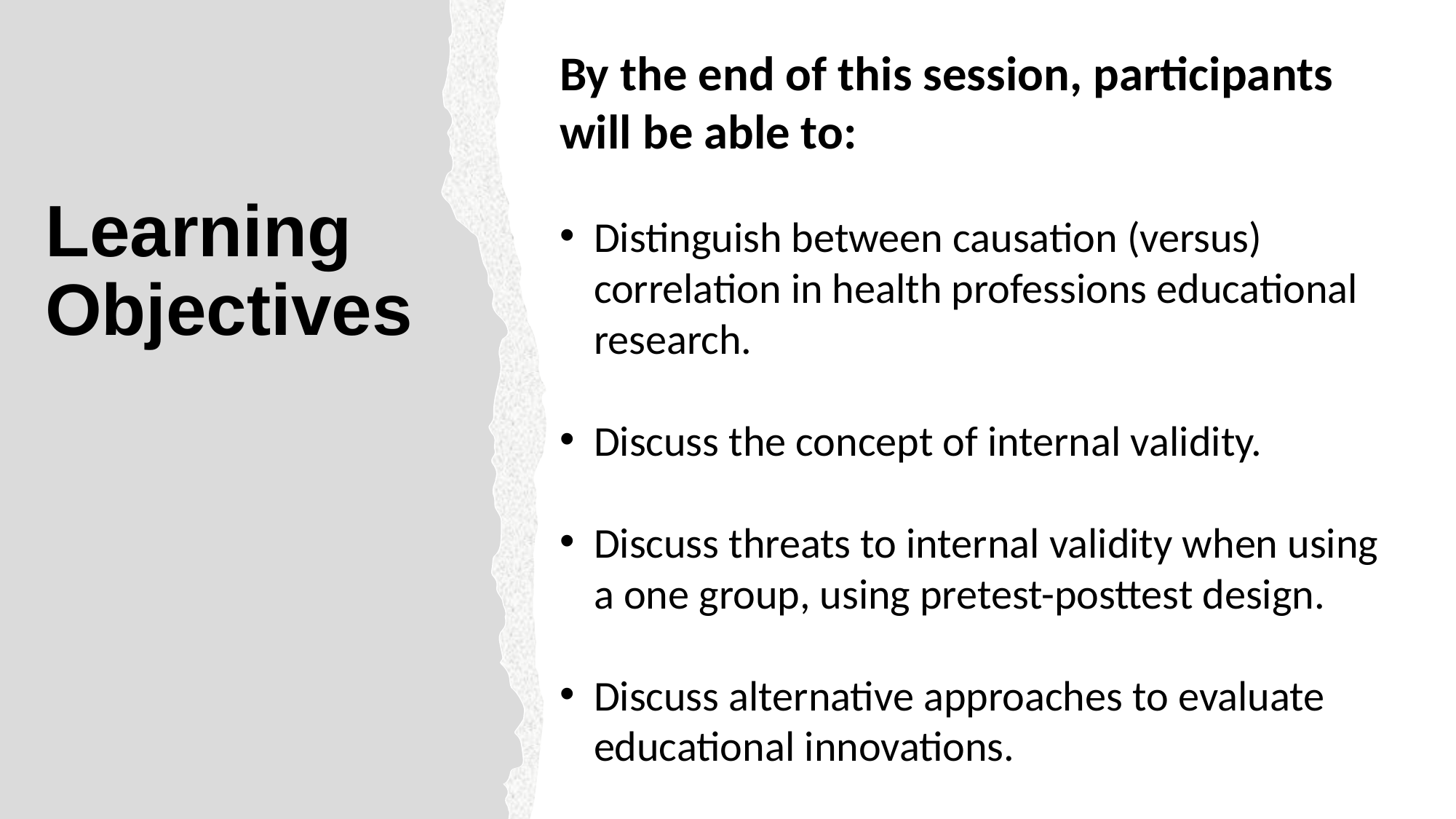

# Learning Objectives
By the end of this session, participants will be able to:
Distinguish between causation (versus) correlation in health professions educational research.
Discuss the concept of internal validity.
Discuss threats to internal validity when using a one group, using pretest-posttest design.
Discuss alternative approaches to evaluate educational innovations.

## Slide 5
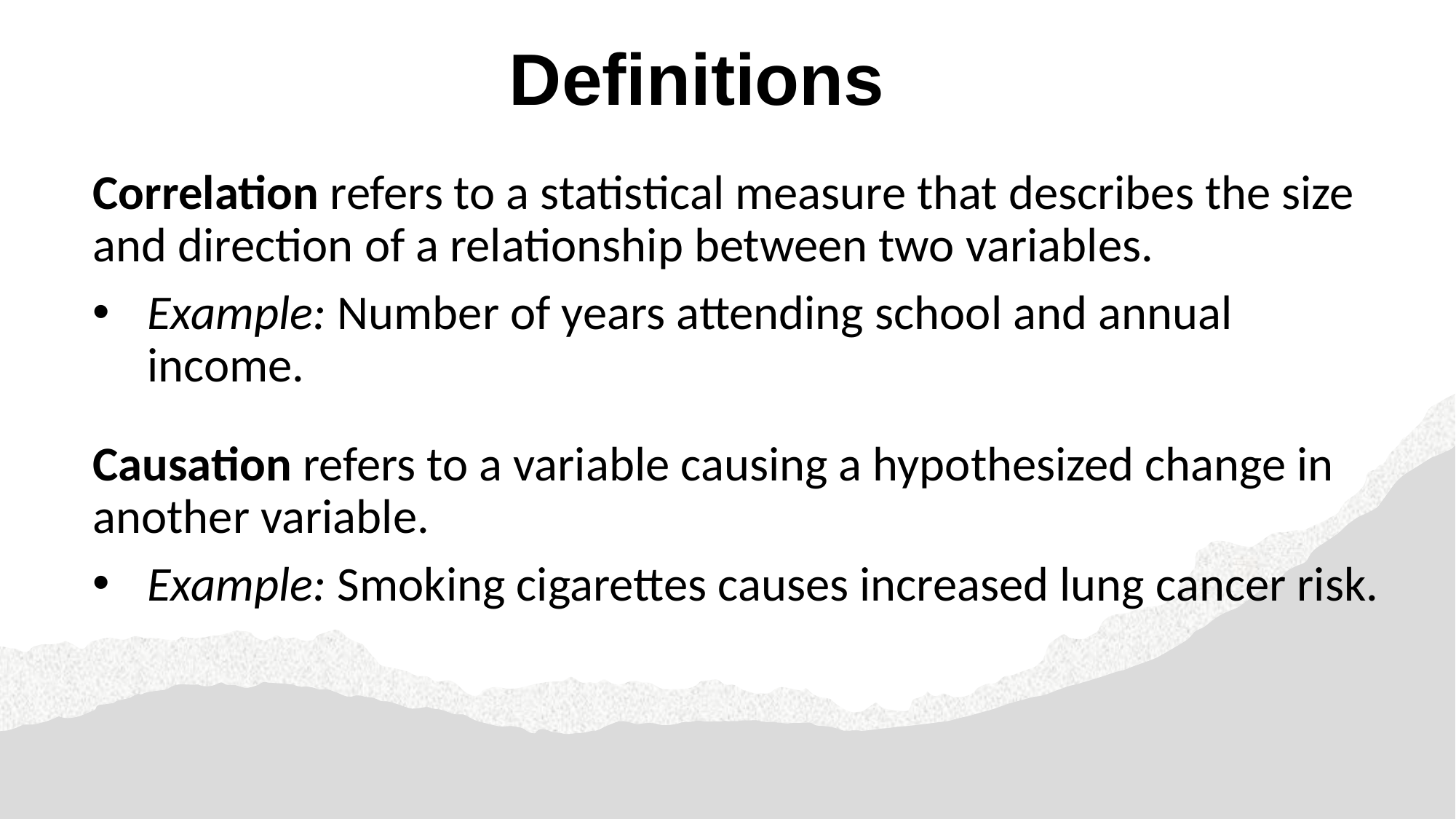

# Definitions
Correlation refers to a statistical measure that describes the size and direction of a relationship between two variables.
Example: Number of years attending school and annual income.
Causation refers to a variable causing a hypothesized change in another variable.
Example: Smoking cigarettes causes increased lung cancer risk.

## Slide 6
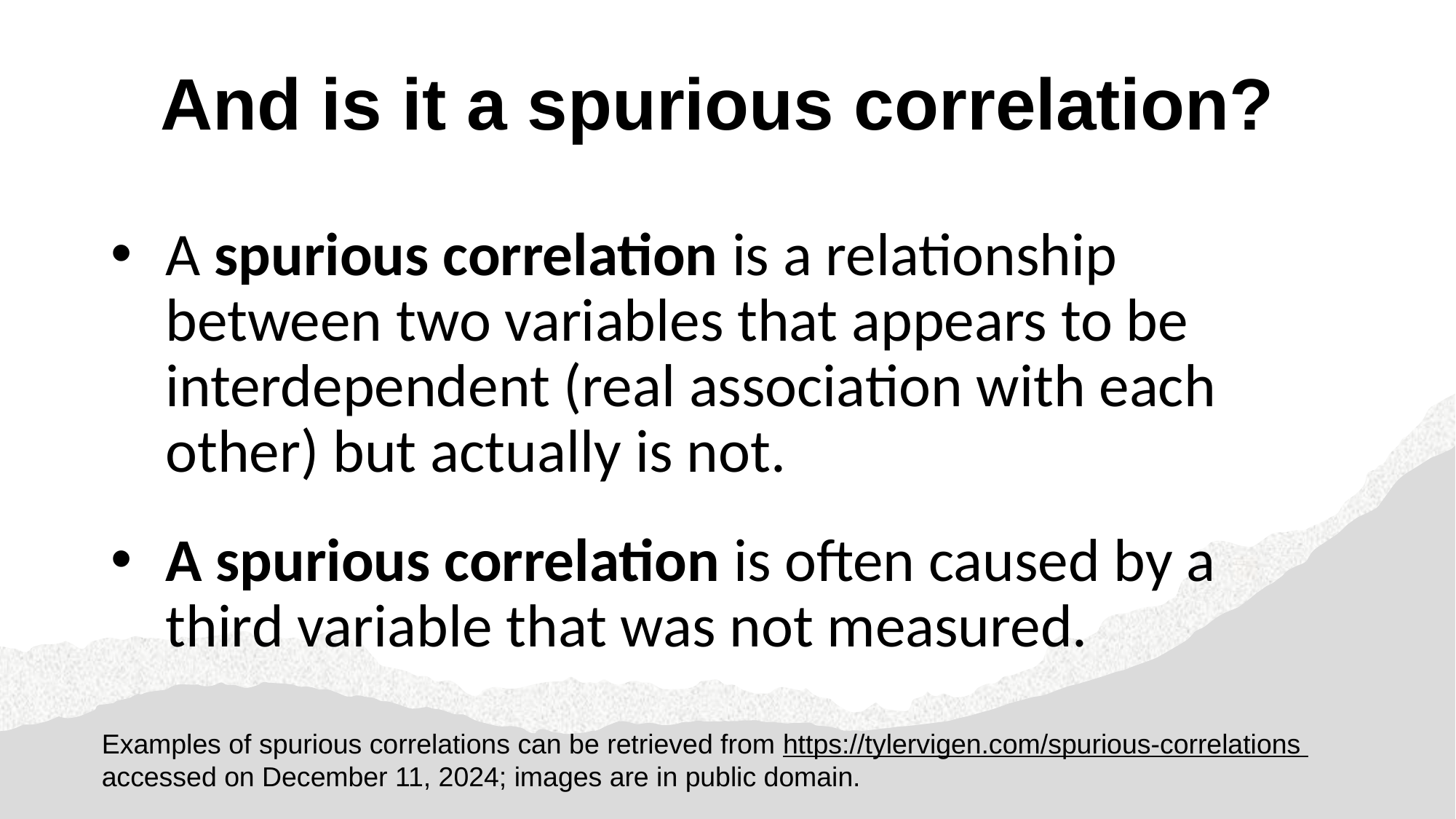

# And is it a spurious correlation?
A spurious correlation is a relationship between two variables that appears to be interdependent (real association with each other) but actually is not.
A spurious correlation is often caused by a third variable that was not measured.
Examples of spurious correlations can be retrieved from https://tylervigen.com/spurious-correlations
accessed on December 11, 2024; images are in public domain.

## Slide 7
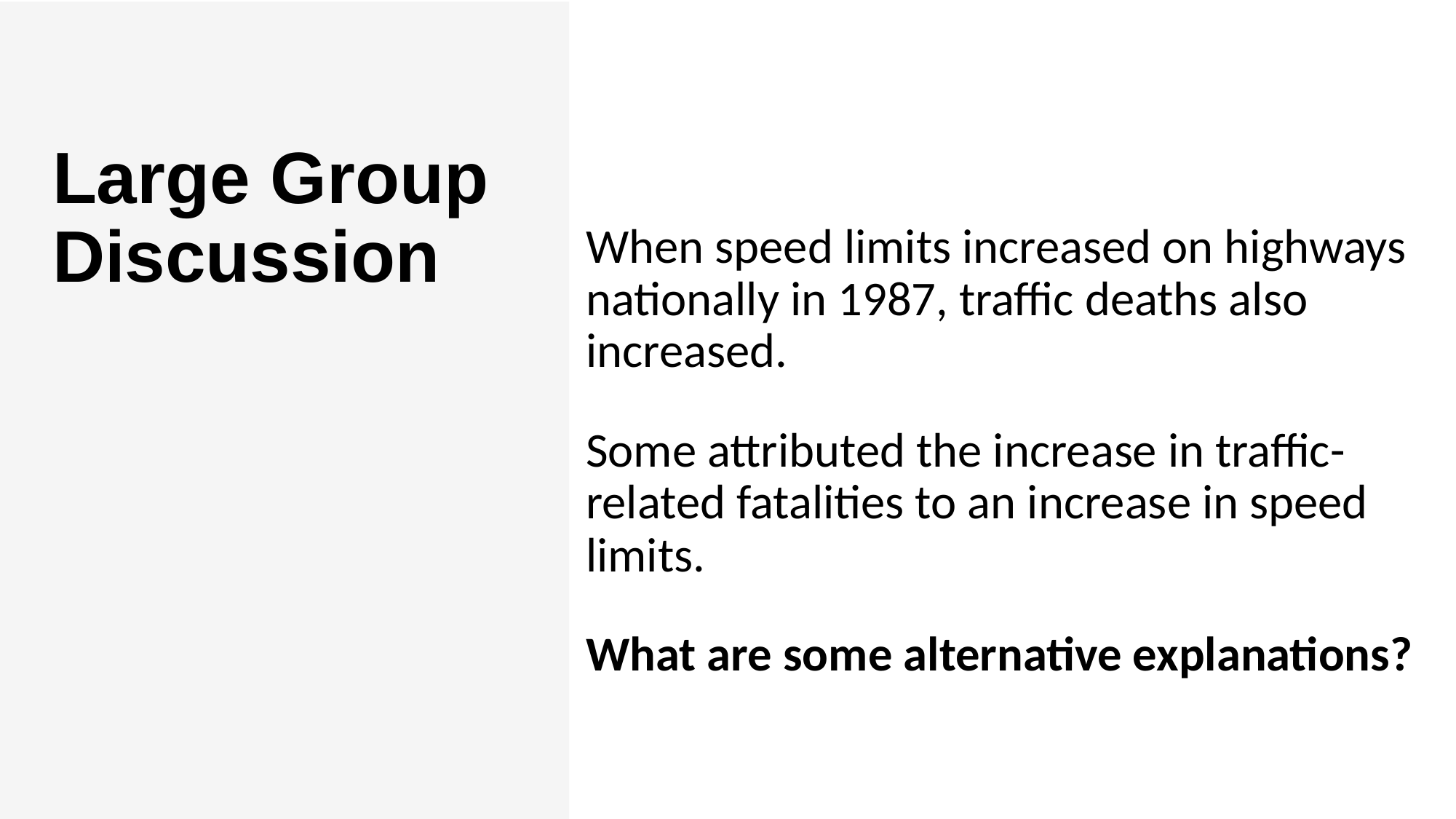

# Large Group Discussion
When speed limits increased on highways nationally in 1987, traffic deaths also increased.
Some attributed the increase in traffic-related fatalities to an increase in speed limits.
What are some alternative explanations?

## Slide 8
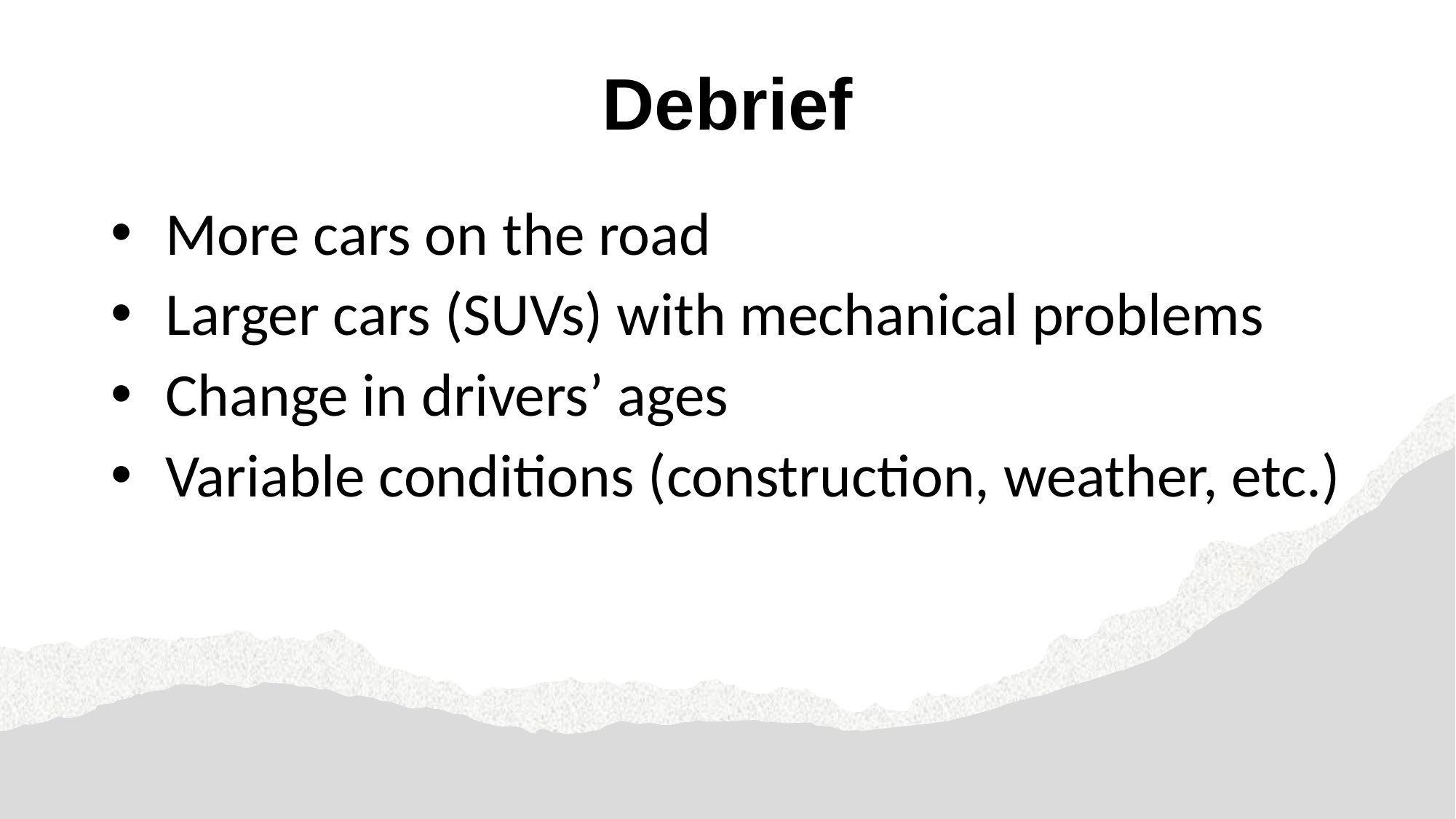

# Debrief
More cars on the road
Larger cars (SUVs) with mechanical problems
Change in drivers’ ages
Variable conditions (construction, weather, etc.)

## Slide 9
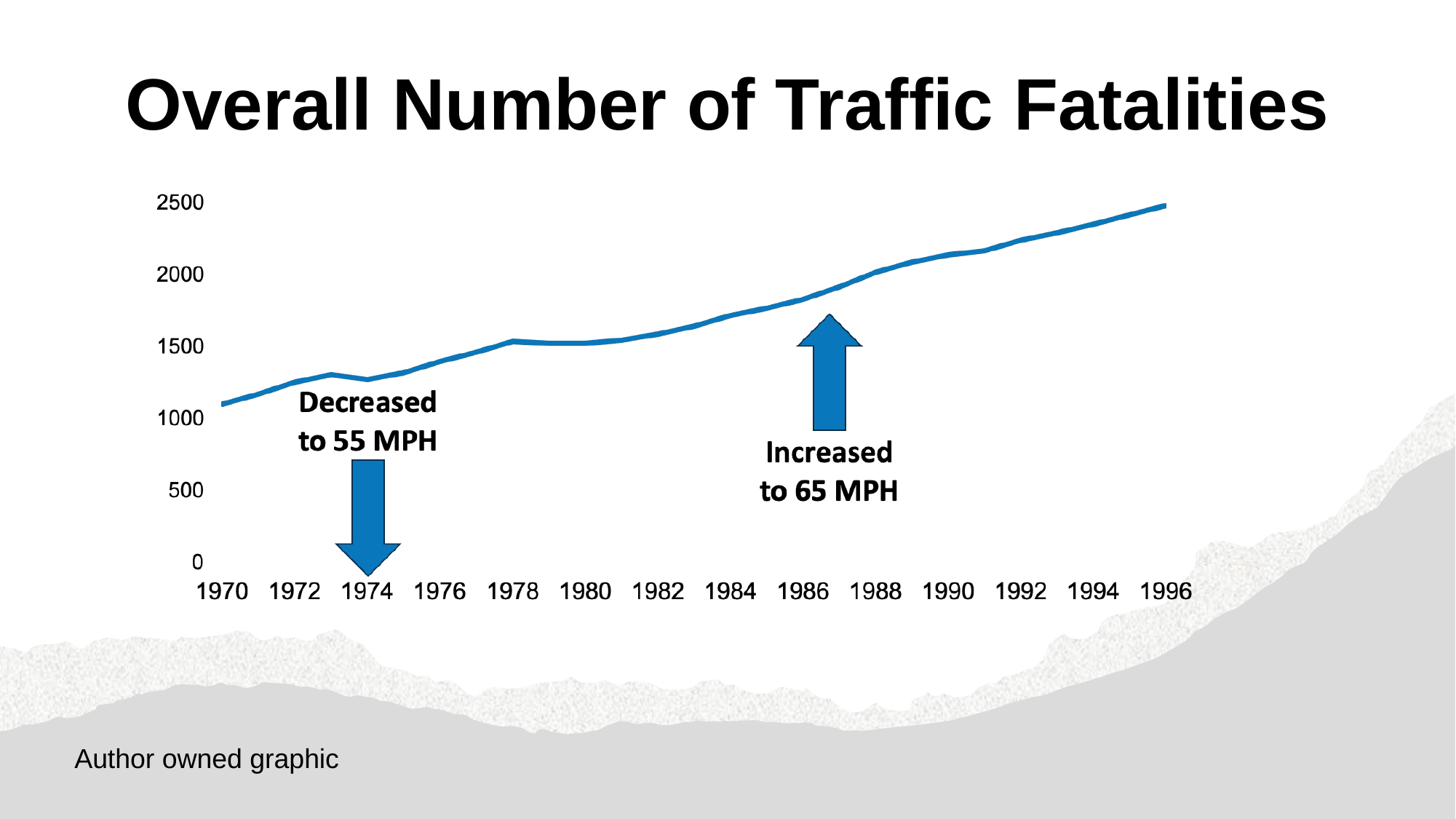

# Overall Number of Traffic Fatalities
Author owned graphic

## Slide 10
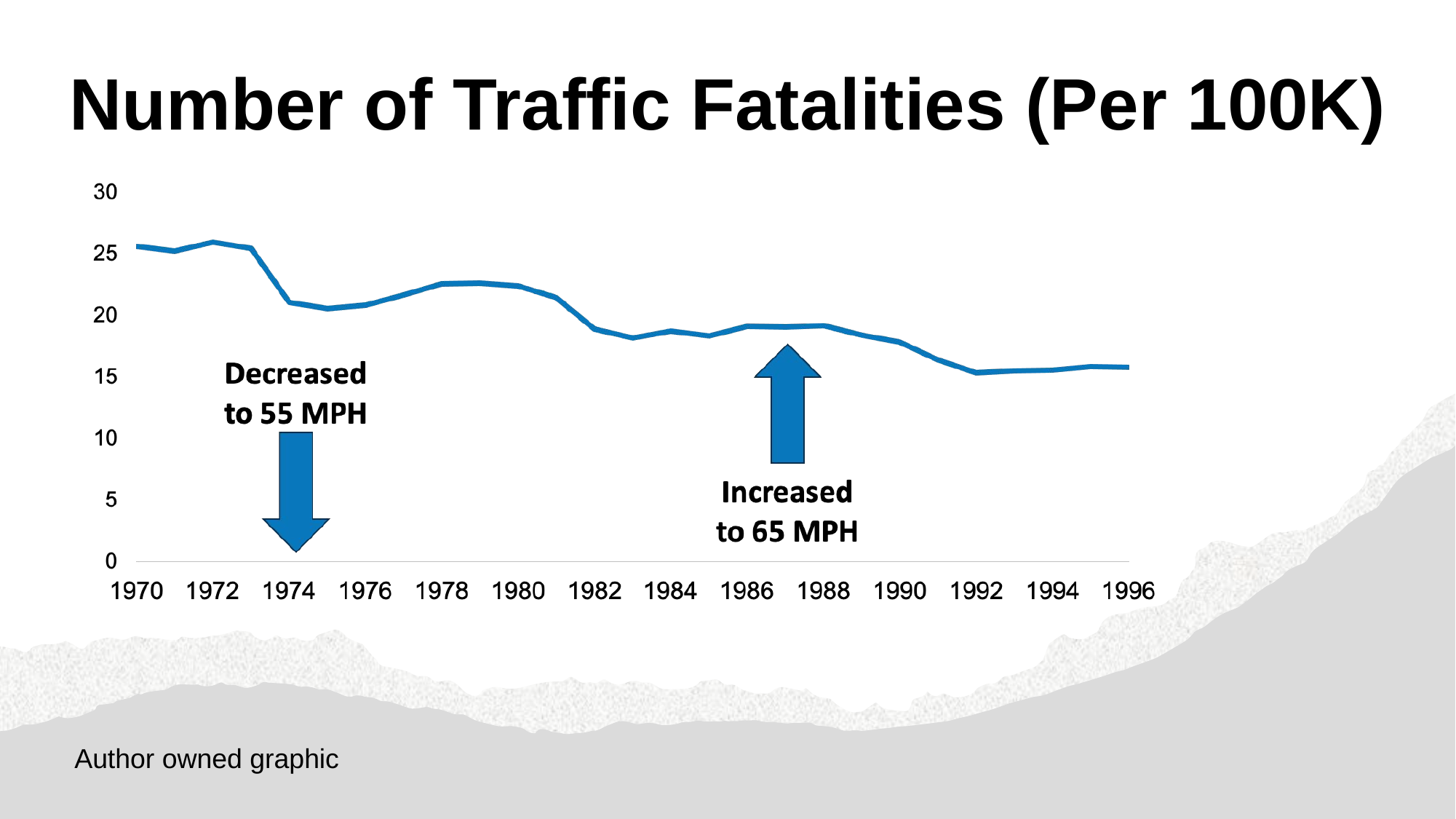

# Number of Traffic Fatalities (Per 100K)
Author owned graphic

## Slide 11
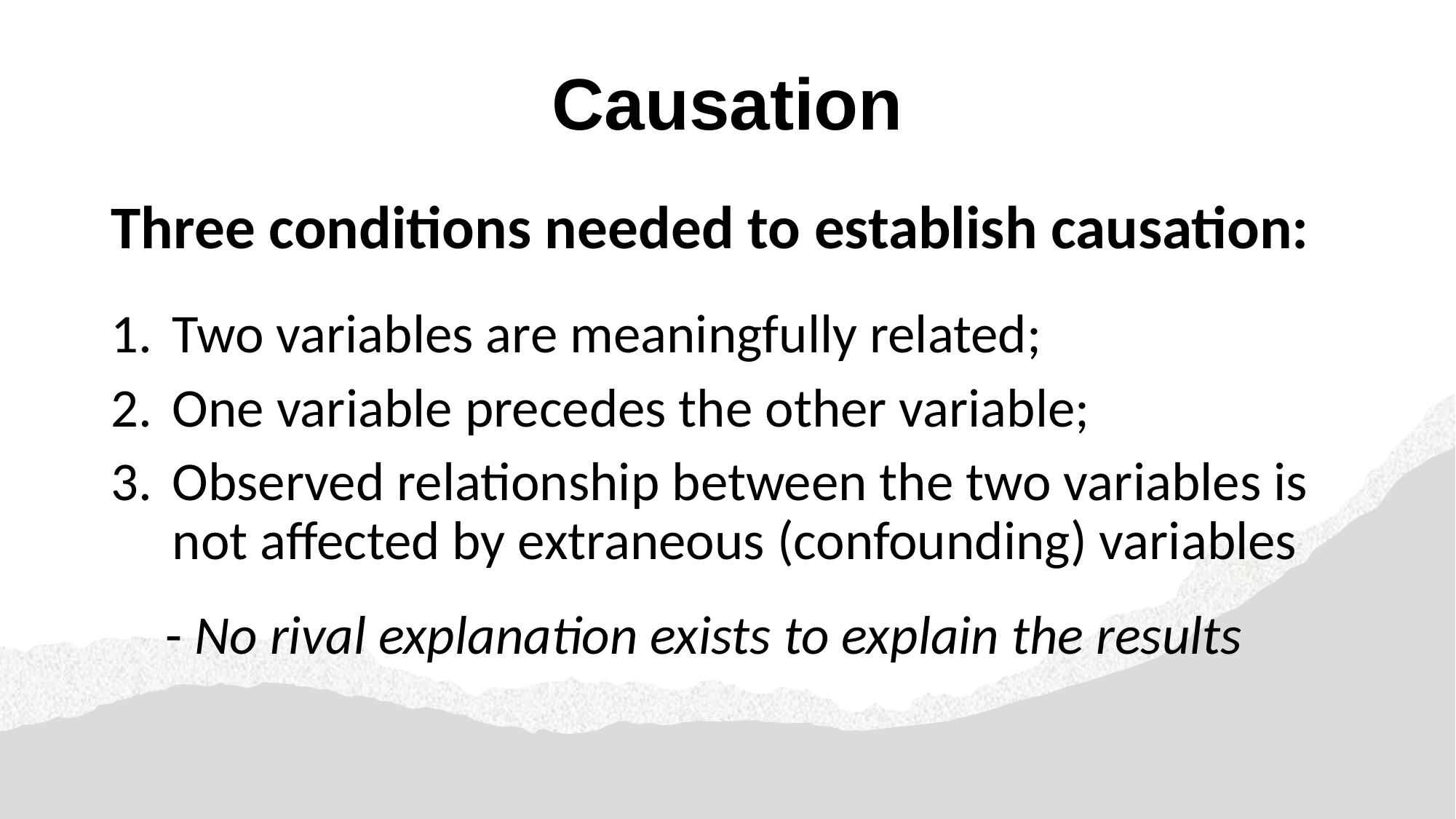

# Causation
Three conditions needed to establish causation:
Two variables are meaningfully related;
One variable precedes the other variable;
Observed relationship between the two variables is not affected by extraneous (confounding) variables
- No rival explanation exists to explain the results

## Slide 12
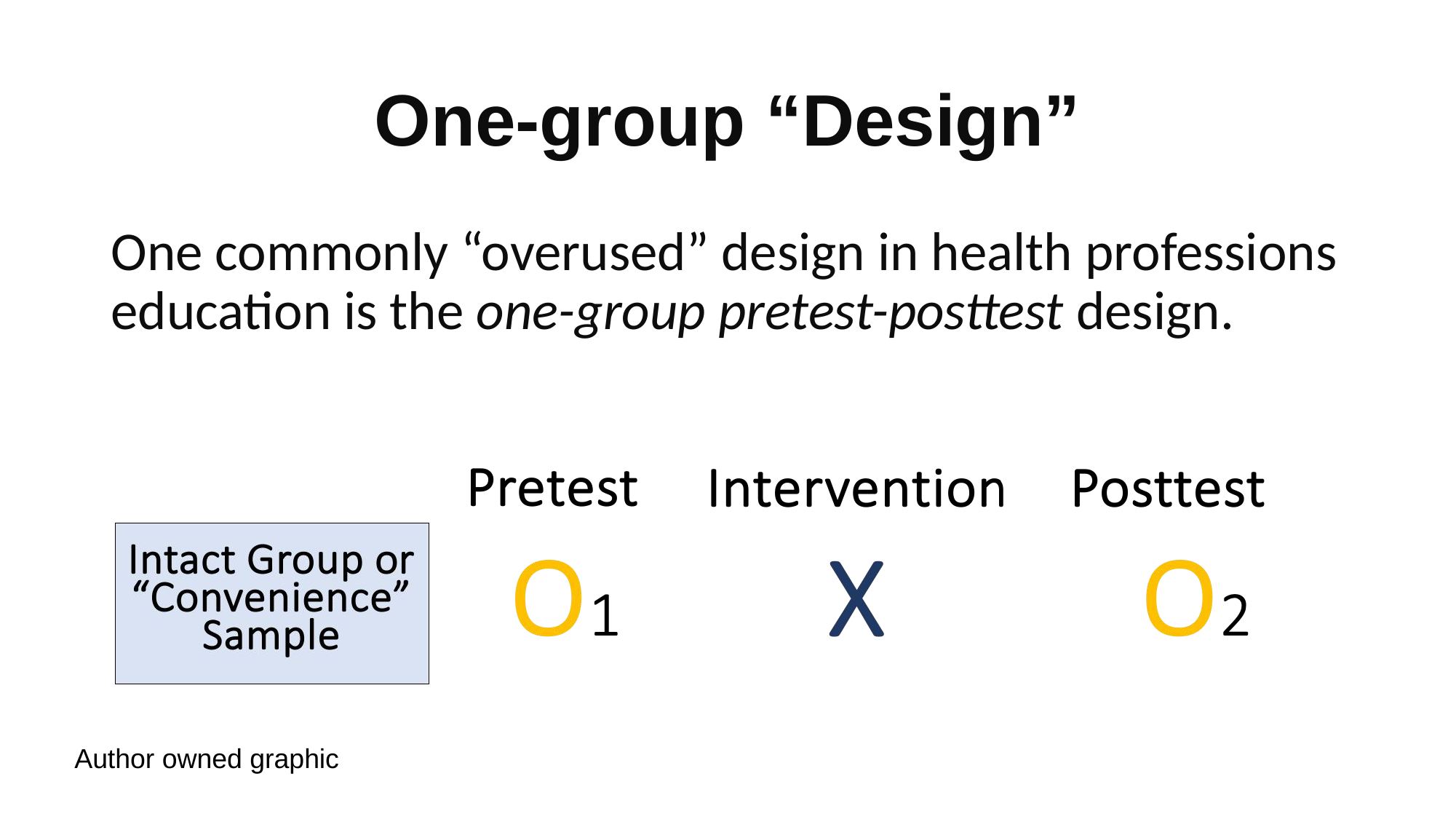

# One-group “Design”
One commonly “overused” design in health professions education is the one-group pretest-posttest design.
Author owned graphic

## Slide 13
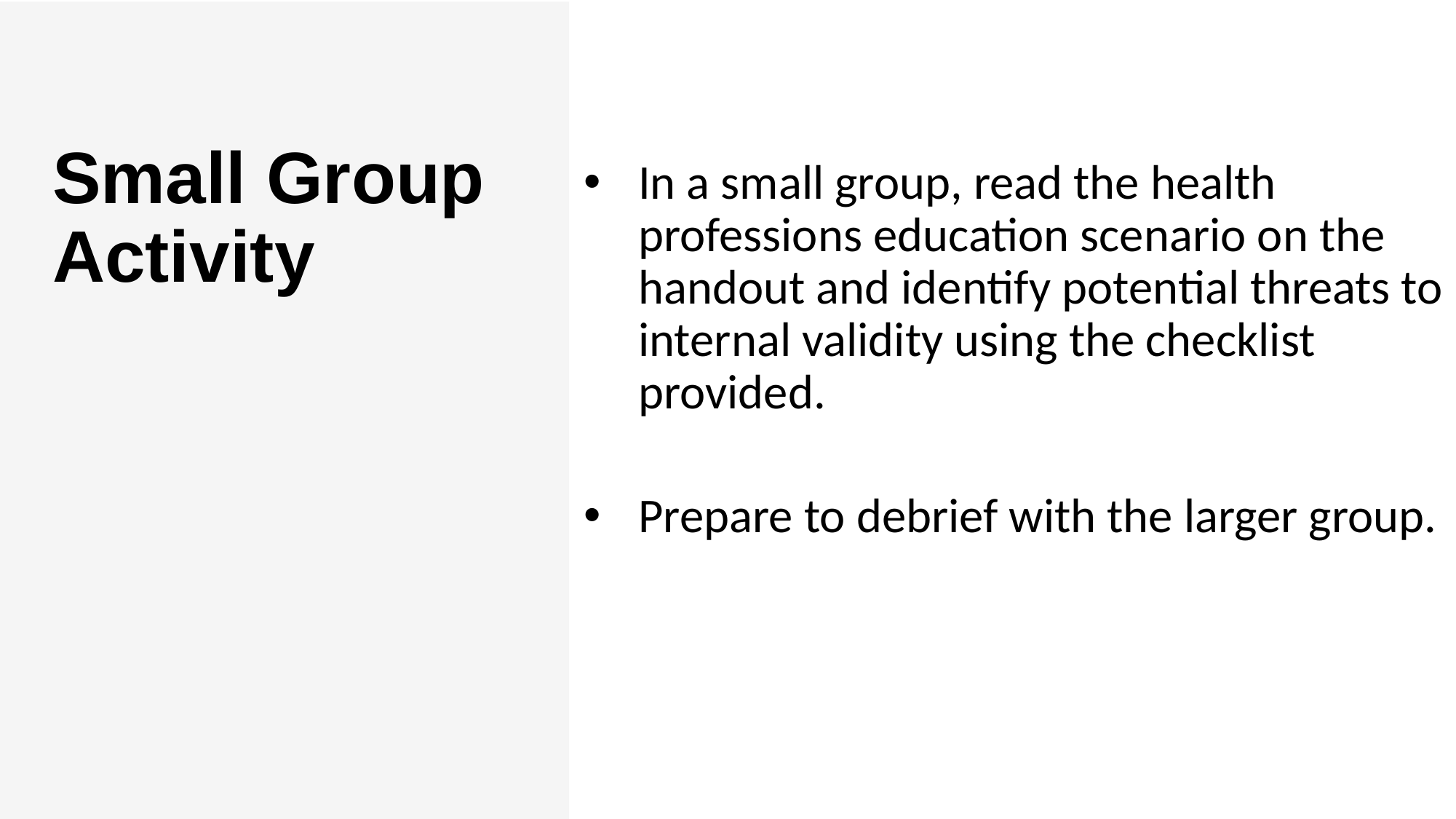

In a small group, read the health professions education scenario on the handout and identify potential threats to internal validity using the checklist provided.
Prepare to debrief with the larger group.
# Small Group Activity

## Slide 14
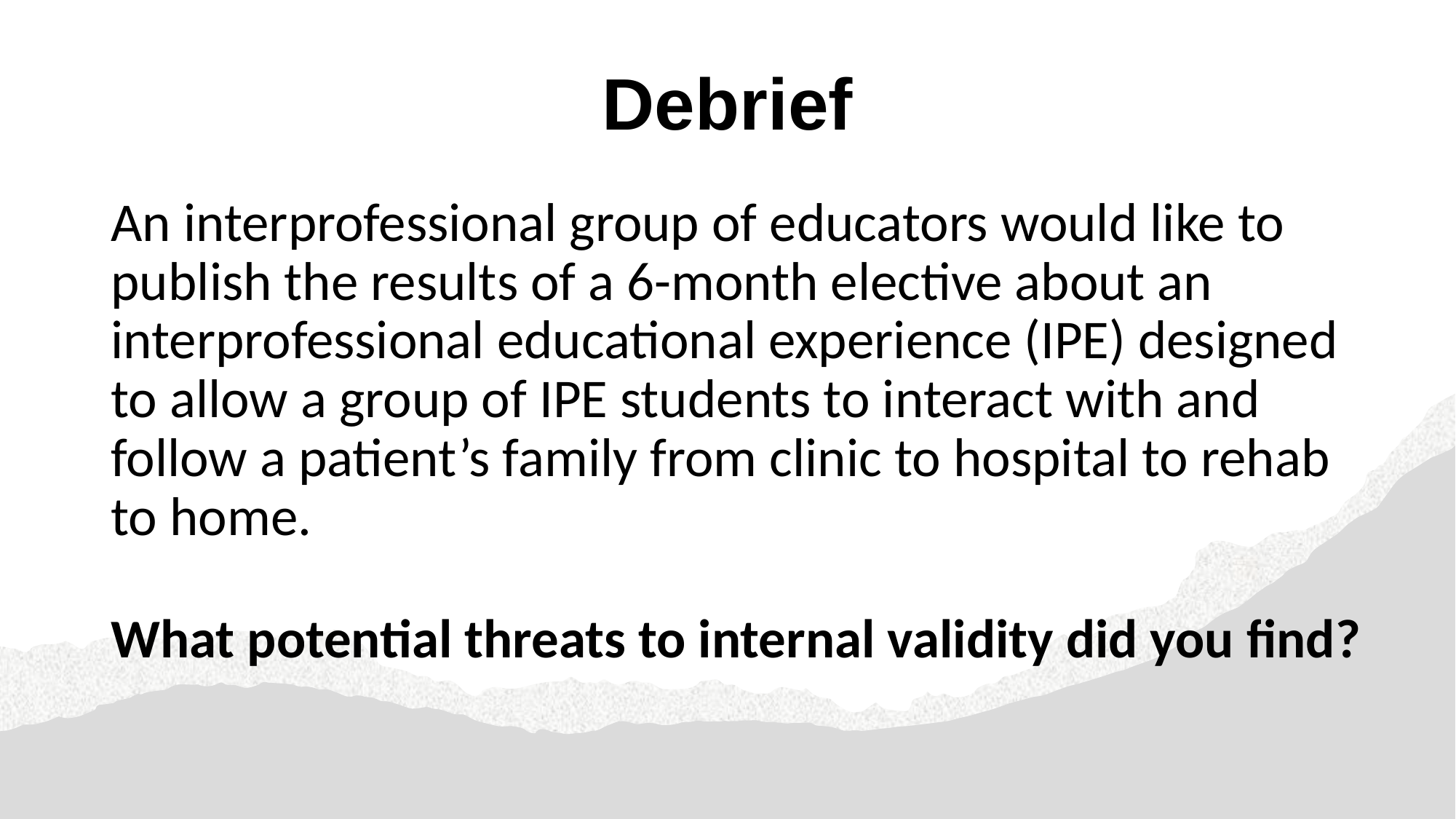

# Debrief
An interprofessional group of educators would like to publish the results of a 6-month elective about an interprofessional educational experience (IPE) designed to allow a group of IPE students to interact with and follow a patient’s family from clinic to hospital to rehab to home.
What potential threats to internal validity did you find?

## Slide 15
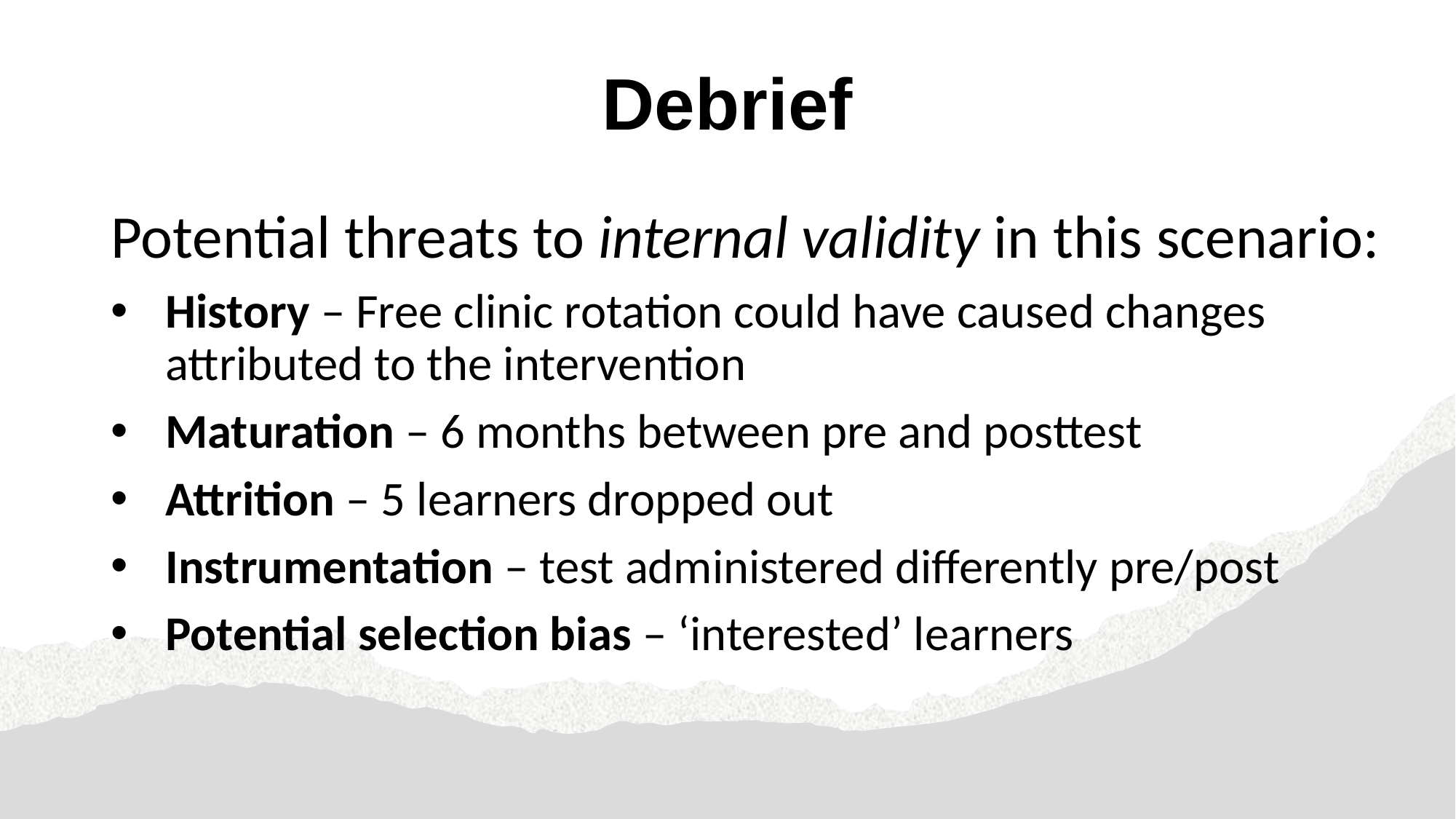

# Debrief
Potential threats to internal validity in this scenario:
History – Free clinic rotation could have caused changes attributed to the intervention
Maturation – 6 months between pre and posttest
Attrition – 5 learners dropped out
Instrumentation – test administered differently pre/post
Potential selection bias – ‘interested’ learners
Potential threats to internal validity in this scenario:
History – Free clinic rotation could have caused changes attributed to the intervention
Maturation – 6 months between pre and posttest
Attrition – 2 students dropped out
Instrumentation – test administered differently pre/post
Potential selection bias – ‘interested’ students

## Slide 16
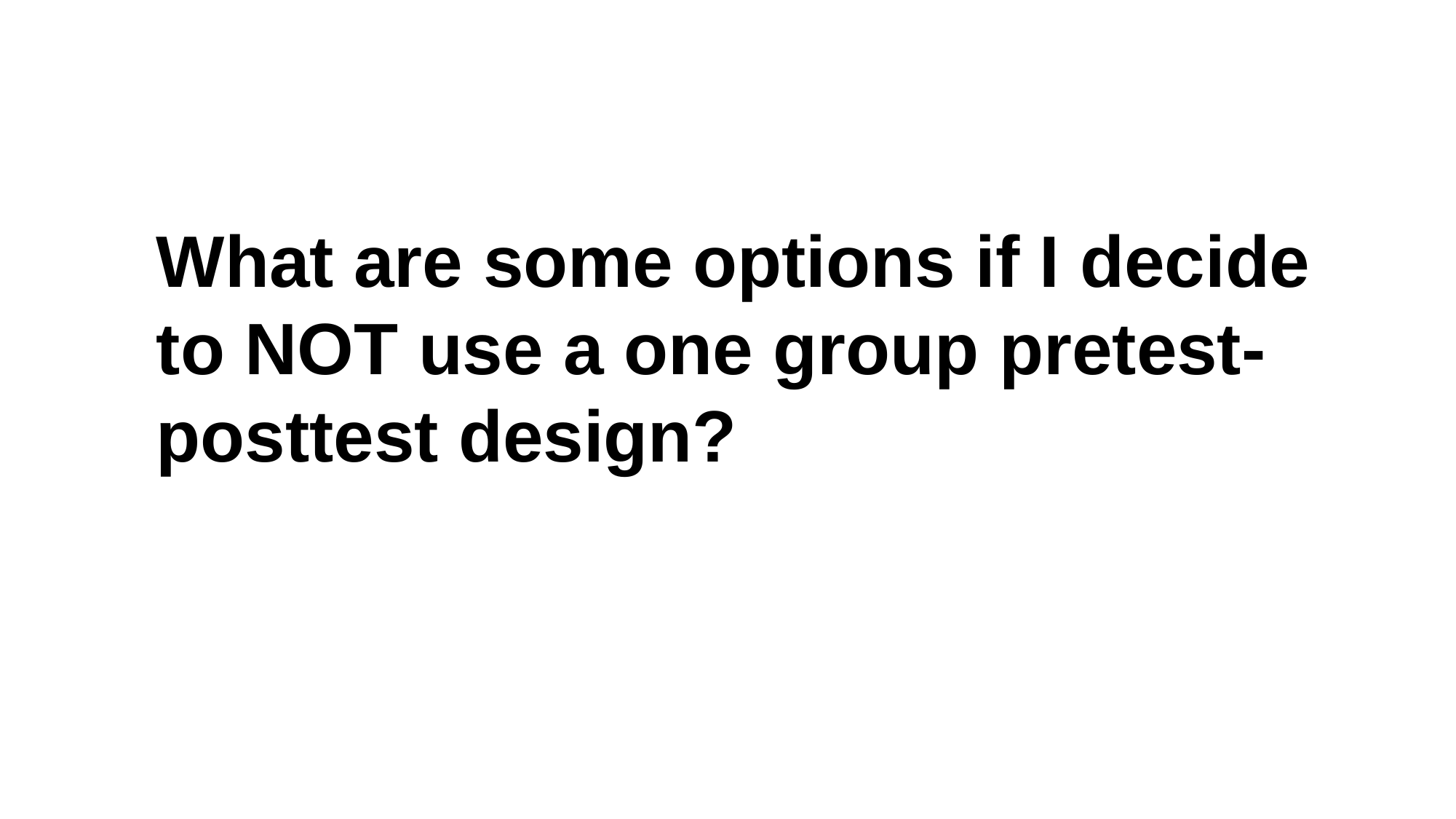

What are some options if I decide to NOT use a one group pretest-posttest design?

## Slide 17
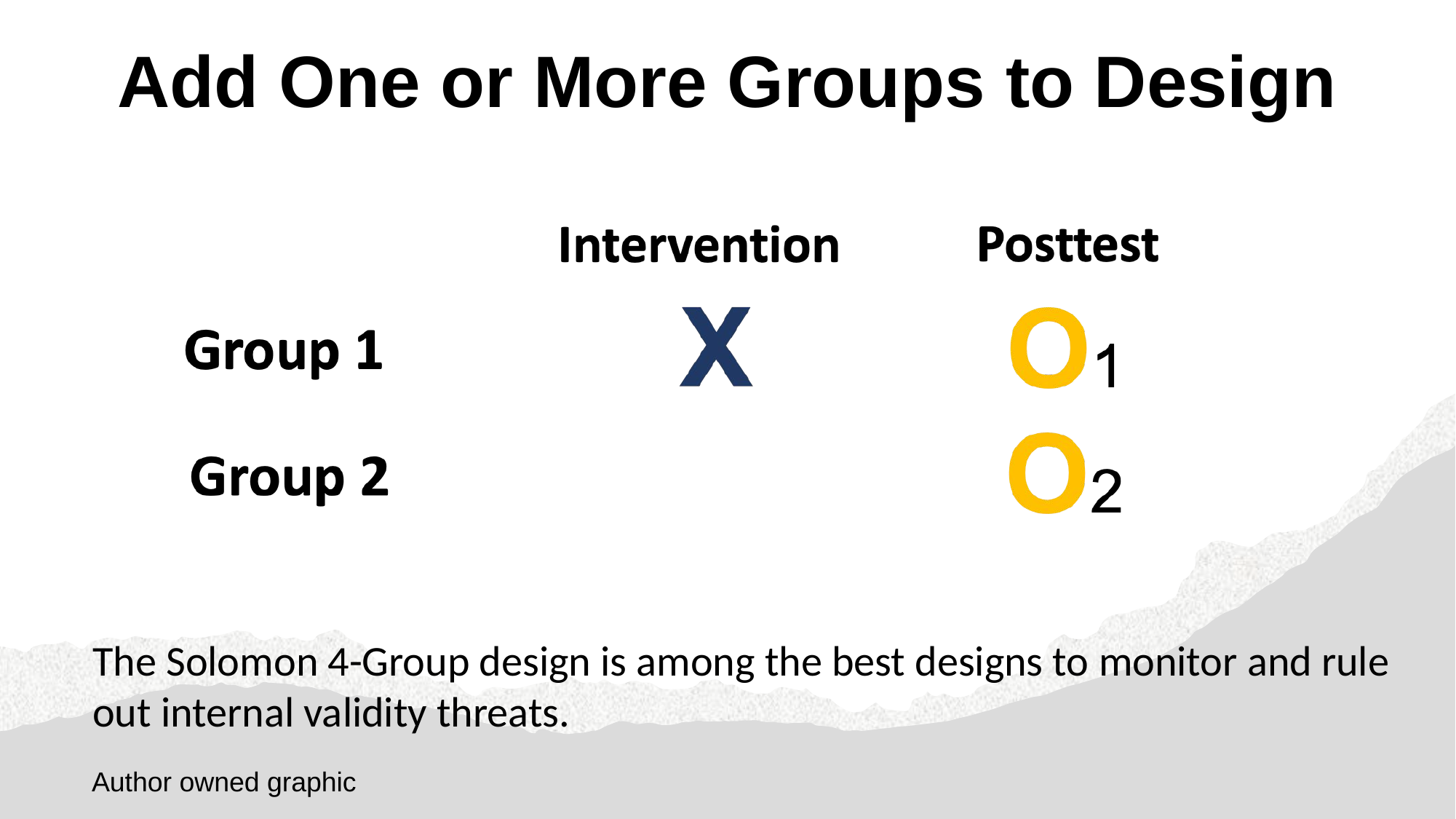

# Add One or More Groups to Design
The Solomon 4-Group design is among the best designs to monitor and rule out internal validity threats.
Author owned graphic

## Slide 18
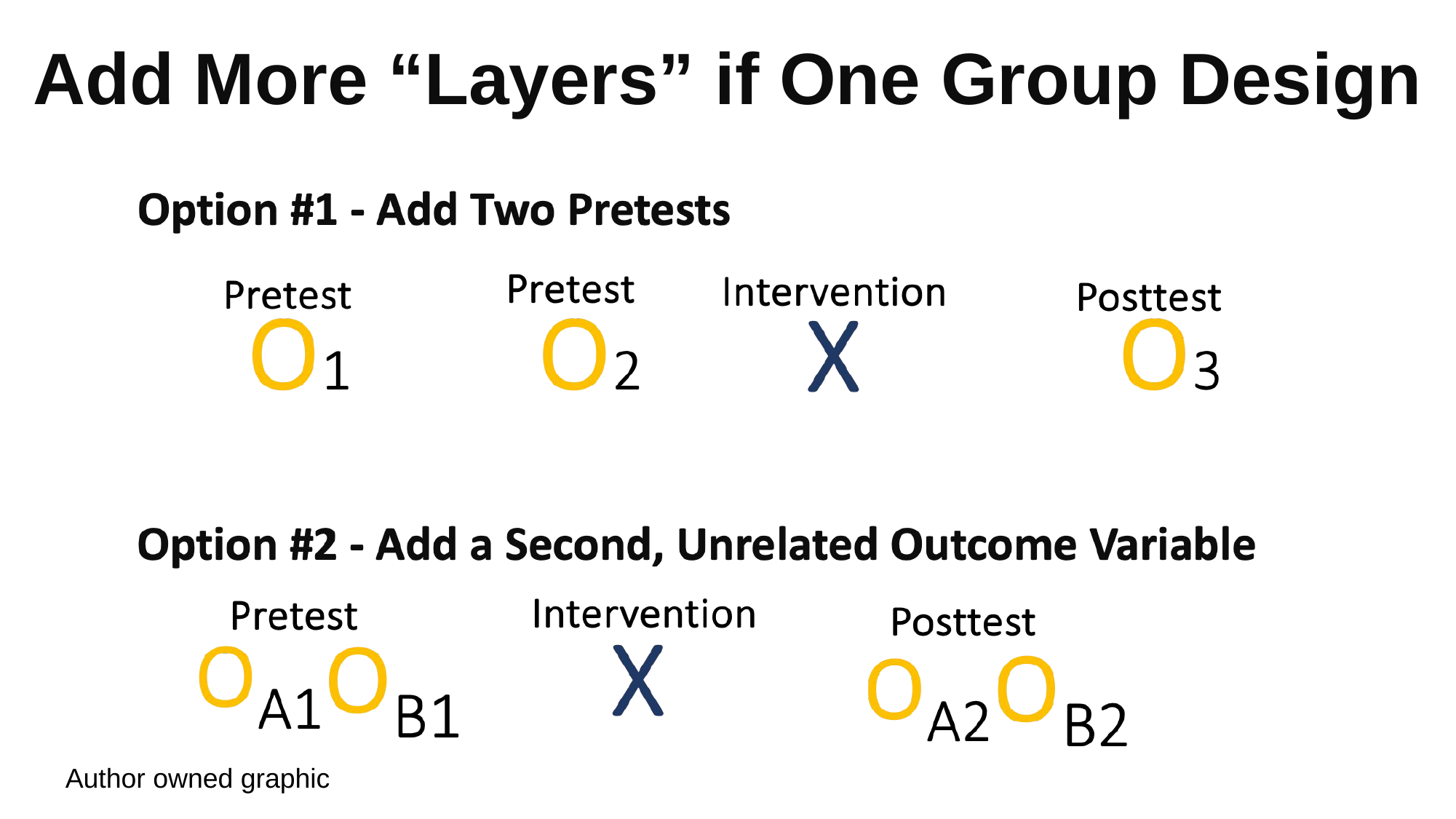

# Add More “Layers” if One Group Design
Author owned graphic

## Slide 19
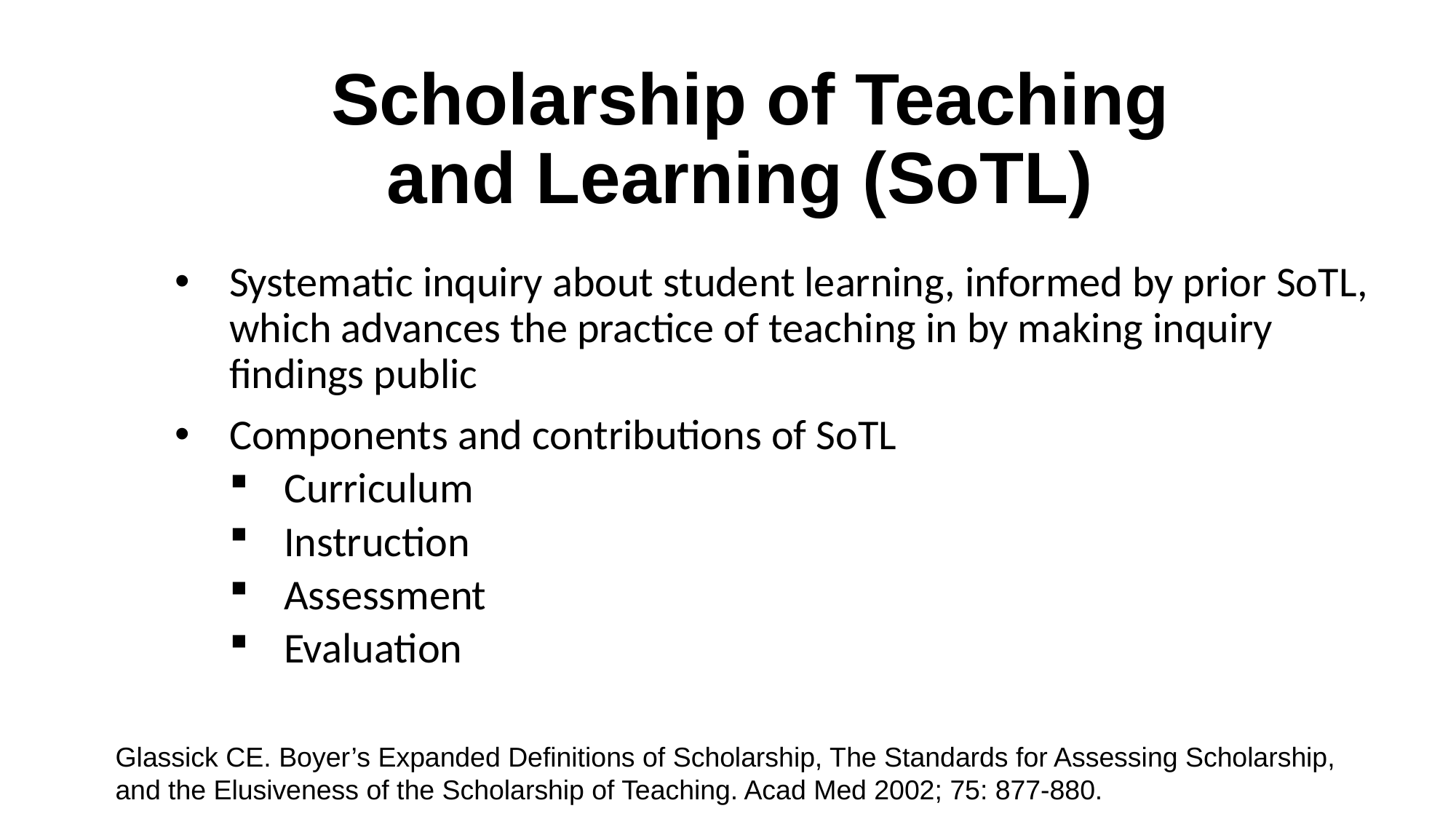

Scholarship of Teaching and Learning (SoTL)
Systematic inquiry about student learning, informed by prior SoTL, which advances the practice of teaching in by making inquiry findings public
Components and contributions of SoTL
Curriculum
Instruction
Assessment
Evaluation
Glassick CE. Boyer’s Expanded Definitions of Scholarship, The Standards for Assessing Scholarship, and the Elusiveness of the Scholarship of Teaching. Acad Med 2002; 75: 877-880.

## Slide 20
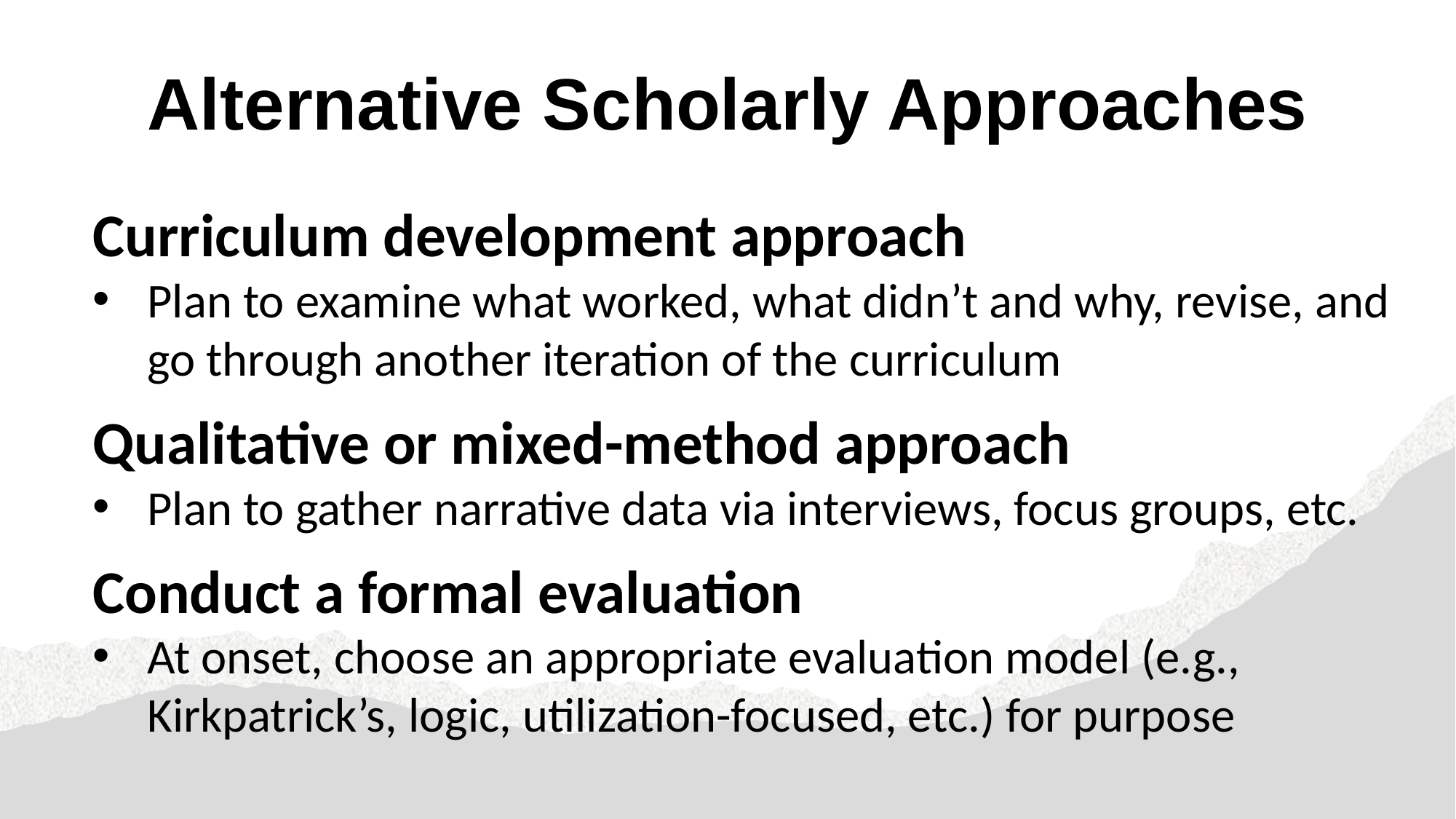

# Alternative Scholarly Approaches
Curriculum development approach
Plan to examine what worked, what didn’t and why, revise, and go through another iteration of the curriculum
Qualitative or mixed-method approach
Plan to gather narrative data via interviews, focus groups, etc.
Conduct a formal evaluation
At onset, choose an appropriate evaluation model (e.g., Kirkpatrick’s, logic, utilization-focused, etc.) for purpose
Potential threats to internal validity in this scenario:
History – Free clinic rotation could have caused changes attributed to the intervention
Maturation – 6 months between pre and posttest
Attrition – 2 students dropped out
Instrumentation – test administered differently pre/post
Potential selection bias – ‘interested’ students

## Slide 21
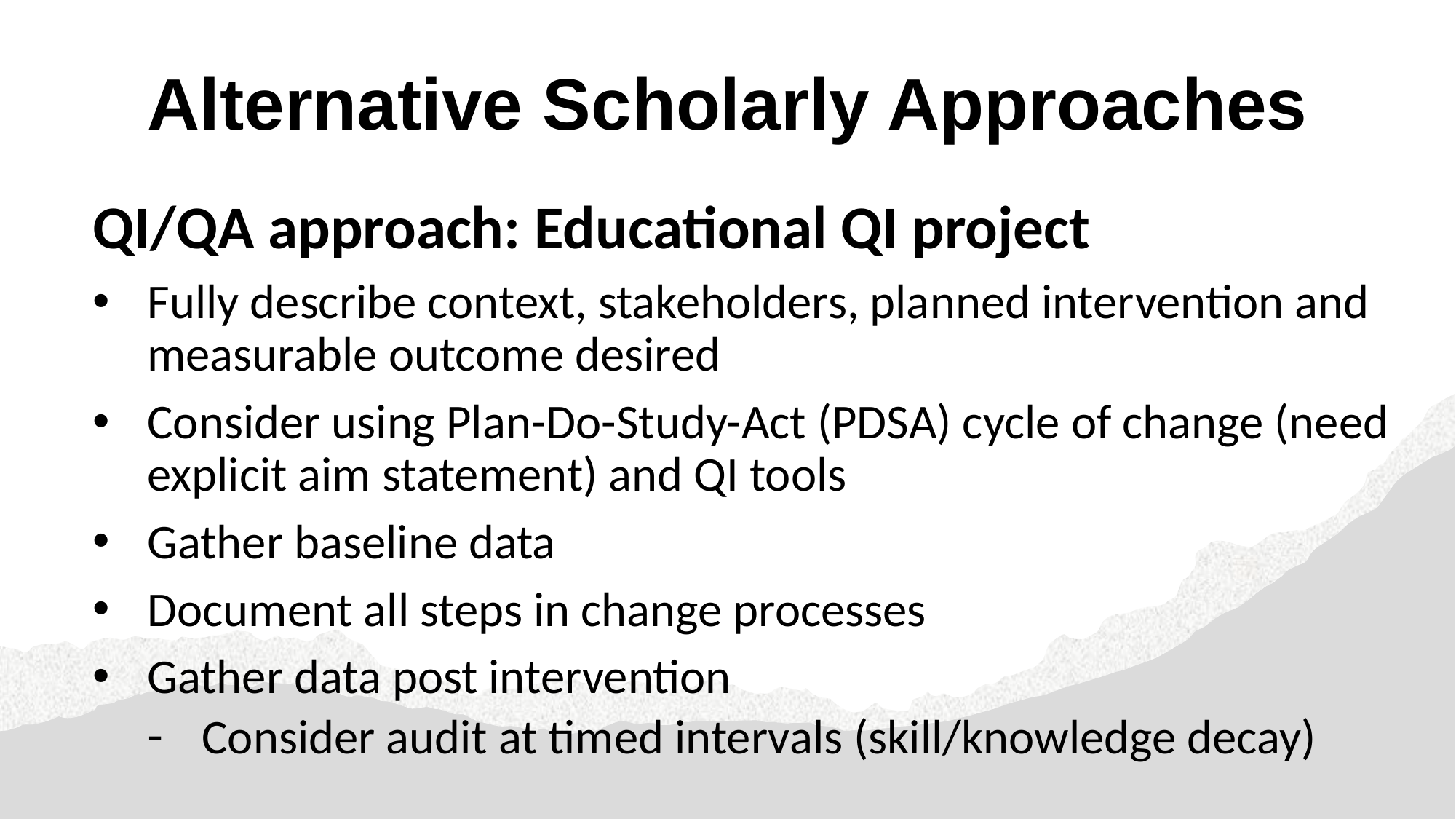

# Alternative Scholarly Approaches
QI/QA approach: Educational QI project
Fully describe context, stakeholders, planned intervention and measurable outcome desired
Consider using Plan-Do-Study-Act (PDSA) cycle of change (need explicit aim statement) and QI tools
Gather baseline data
Document all steps in change processes
Gather data post intervention
Consider audit at timed intervals (skill/knowledge decay)
Potential threats to internal validity in this scenario:
History – Free clinic rotation could have caused changes attributed to the intervention
Maturation – 6 months between pre and posttest
Attrition – 2 students dropped out
Instrumentation – test administered differently pre/post
Potential selection bias – ‘interested’ students

## Slide 22
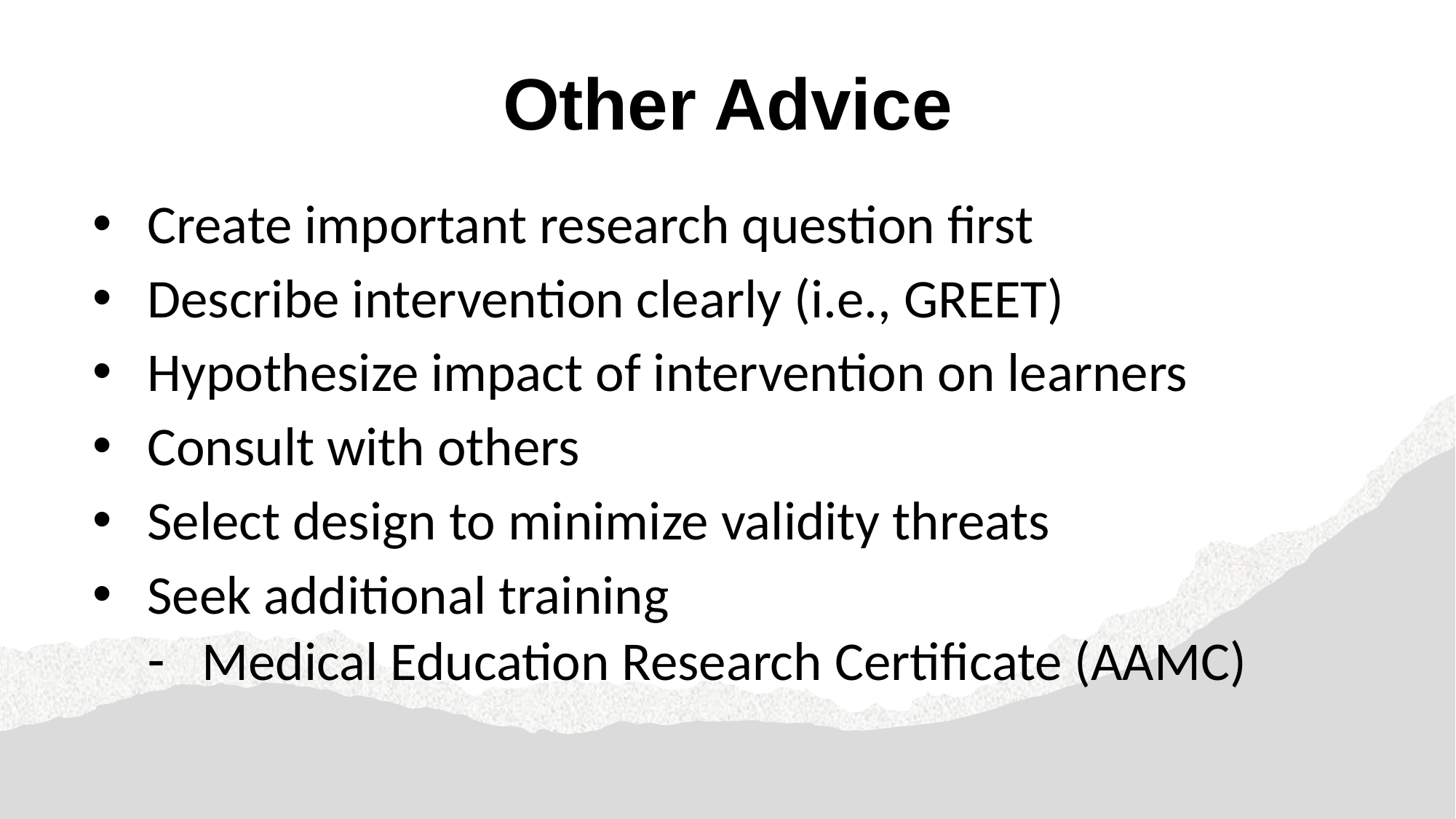

# Other Advice
Create important research question first
Describe intervention clearly (i.e., GREET)
Hypothesize impact of intervention on learners
Consult with others
Select design to minimize validity threats
Seek additional training
Medical Education Research Certificate (AAMC)
Potential threats to internal validity in this scenario:
History – Free clinic rotation could have caused changes attributed to the intervention
Maturation – 6 months between pre and posttest
Attrition – 2 students dropped out
Instrumentation – test administered differently pre/post
Potential selection bias – ‘interested’ students

## Slide 23
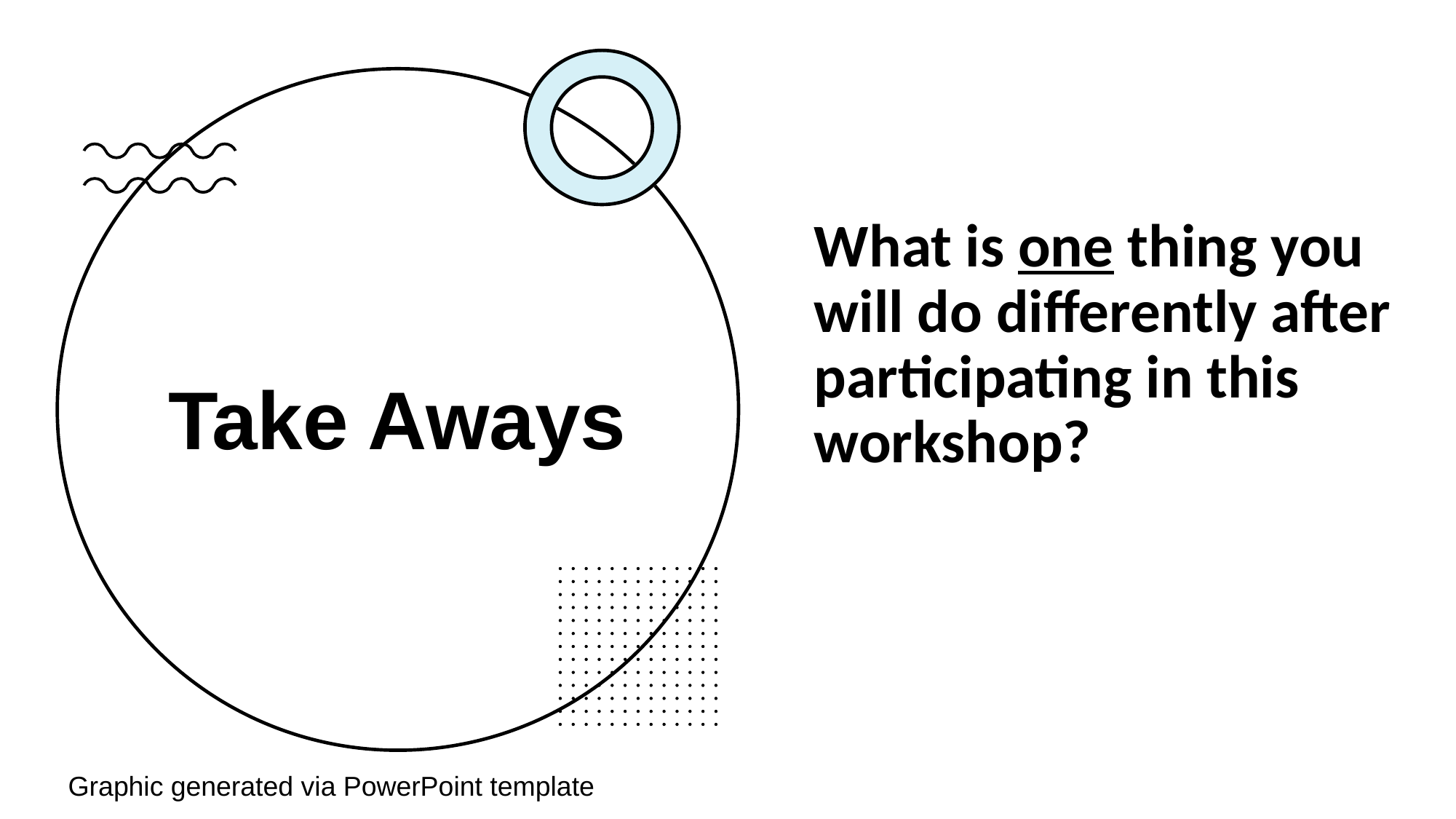

# Take Aways
What is one thing you will do differently after participating in this workshop?
Graphic generated via PowerPoint template

## Slide 24
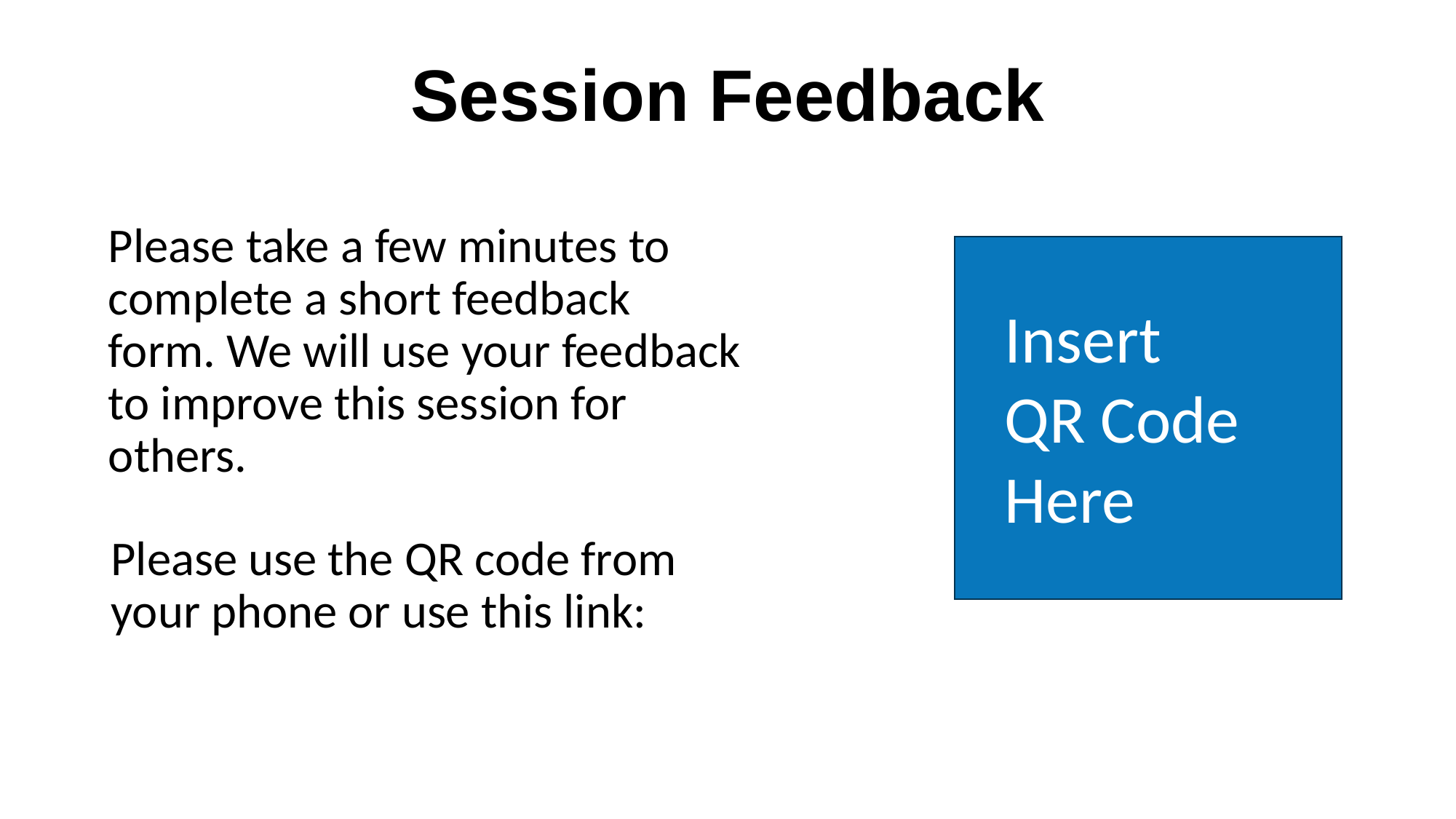

Session Feedback
Please take a few minutes to complete a short feedback form. We will use your feedback to improve this session for others.
Insert
QR Code
Here
Please use the QR code from your phone or use this link:

## Slide 25
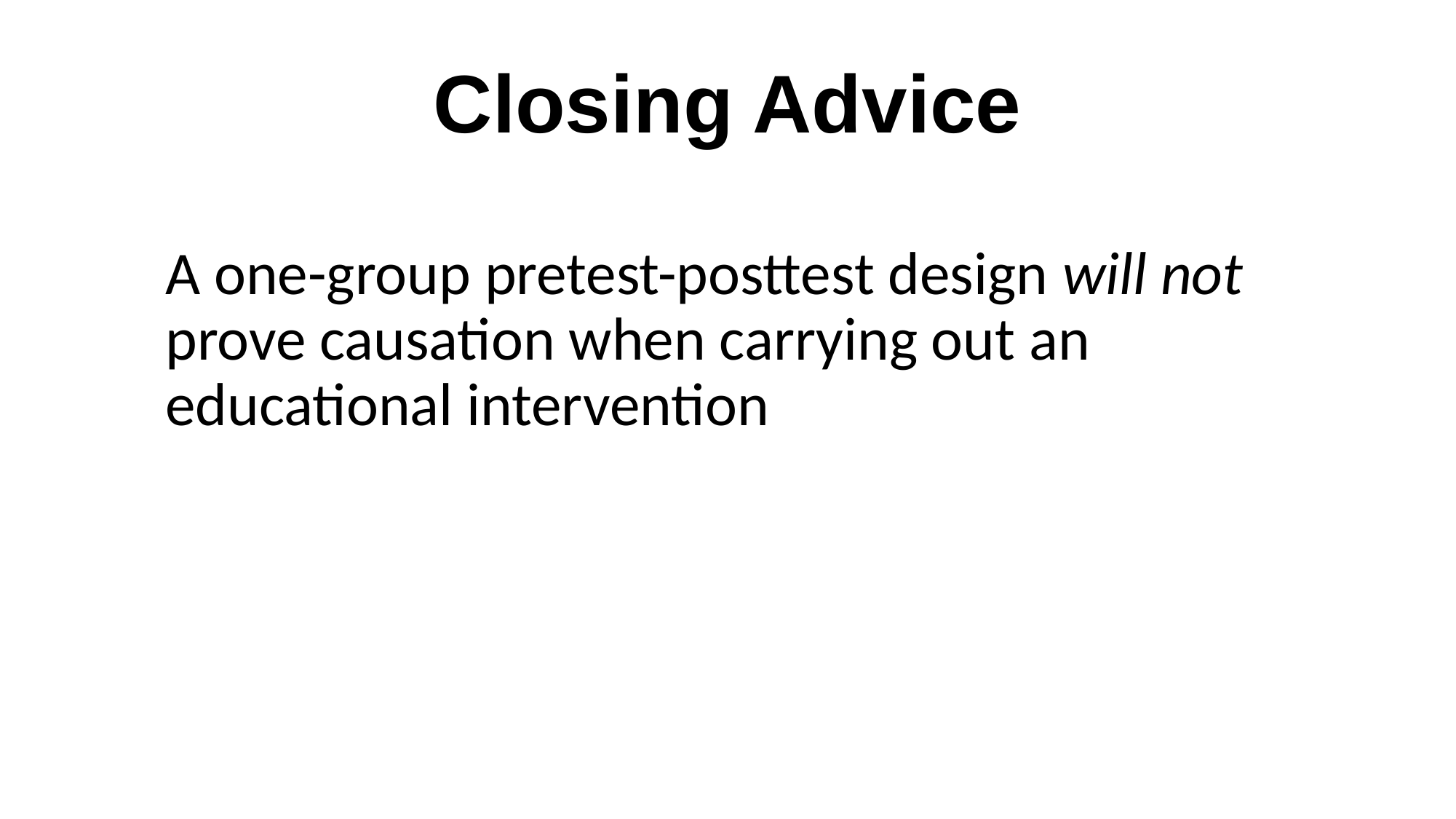

# Closing Advice
A one-group pretest-posttest design will not prove causation when carrying out an educational intervention

## Slide 26
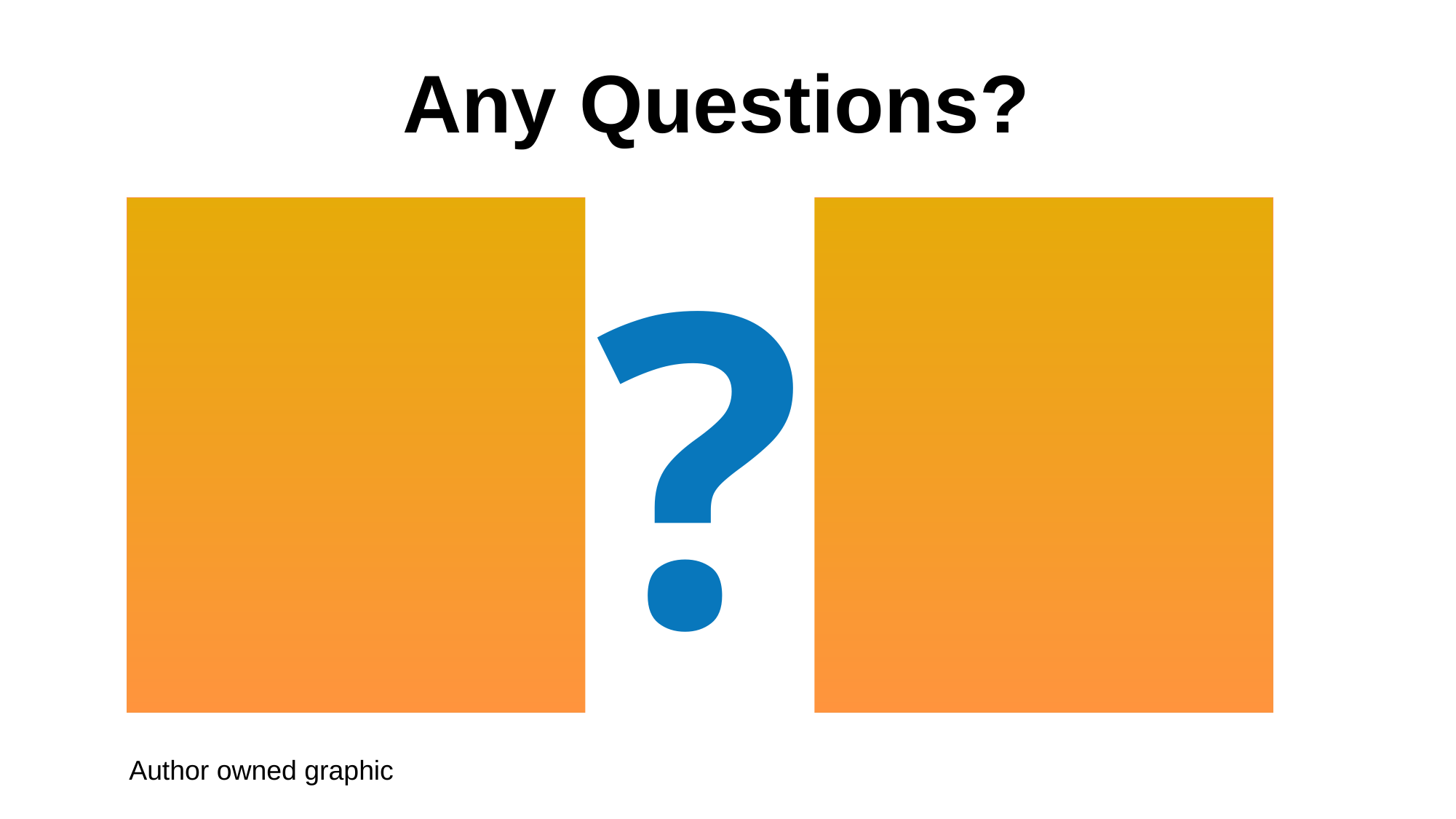

# Any Questions?
?
Author owned graphic

## Slide 27
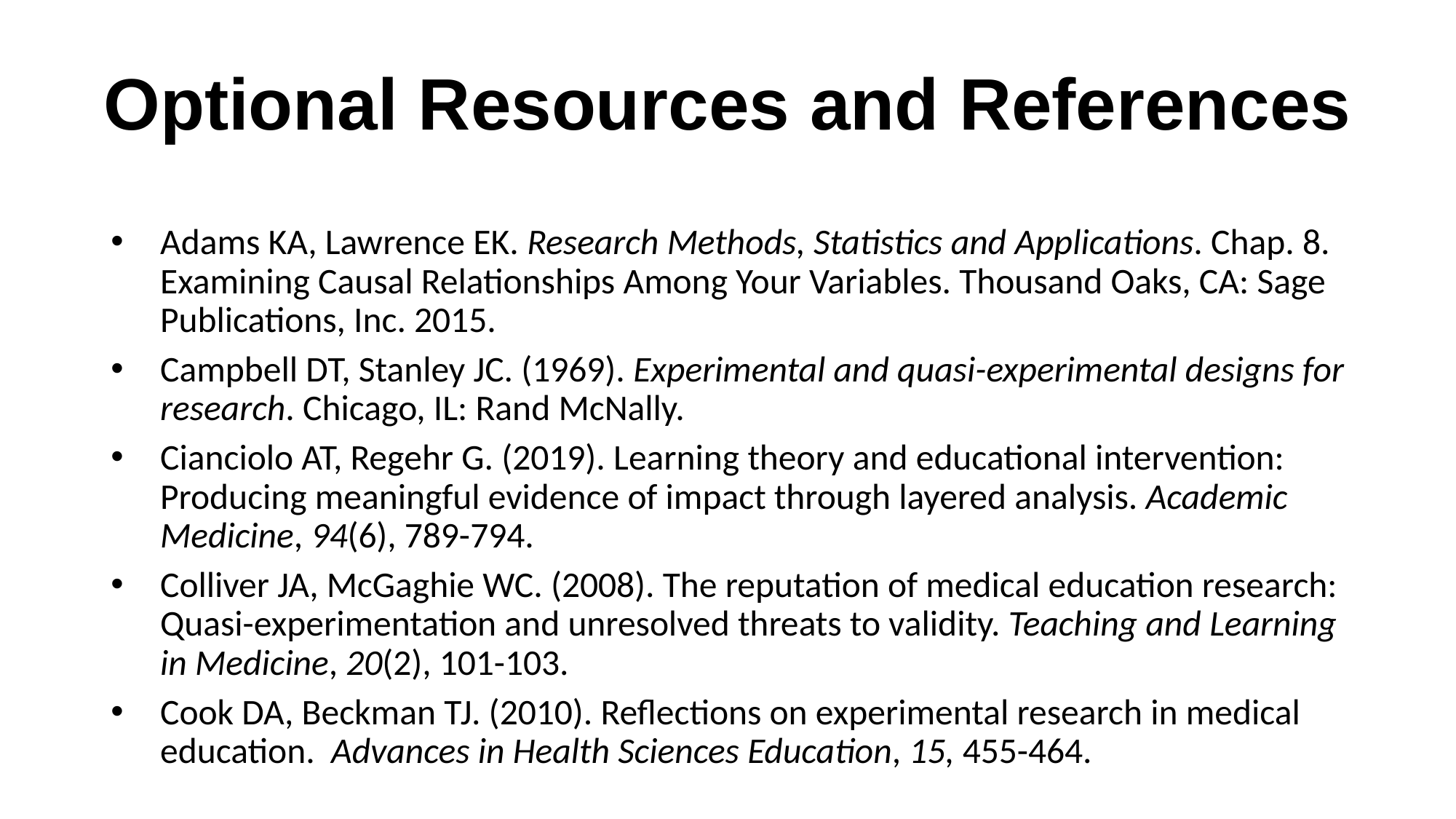

# Optional Resources and References
Adams KA, Lawrence EK. Research Methods, Statistics and Applications. Chap. 8. Examining Causal Relationships Among Your Variables. Thousand Oaks, CA: Sage Publications, Inc. 2015.
Campbell DT, Stanley JC. (1969). Experimental and quasi-experimental designs for research. Chicago, IL: Rand McNally.
Cianciolo AT, Regehr G. (2019). Learning theory and educational intervention: Producing meaningful evidence of impact through layered analysis. Academic Medicine, 94(6), 789-794.
Colliver JA, McGaghie WC. (2008). The reputation of medical education research: Quasi-experimentation and unresolved threats to validity. Teaching and Learning in Medicine, 20(2), 101-103.
Cook DA, Beckman TJ. (2010). Reflections on experimental research in medical education. Advances in Health Sciences Education, 15, 455-464.

## Slide 28
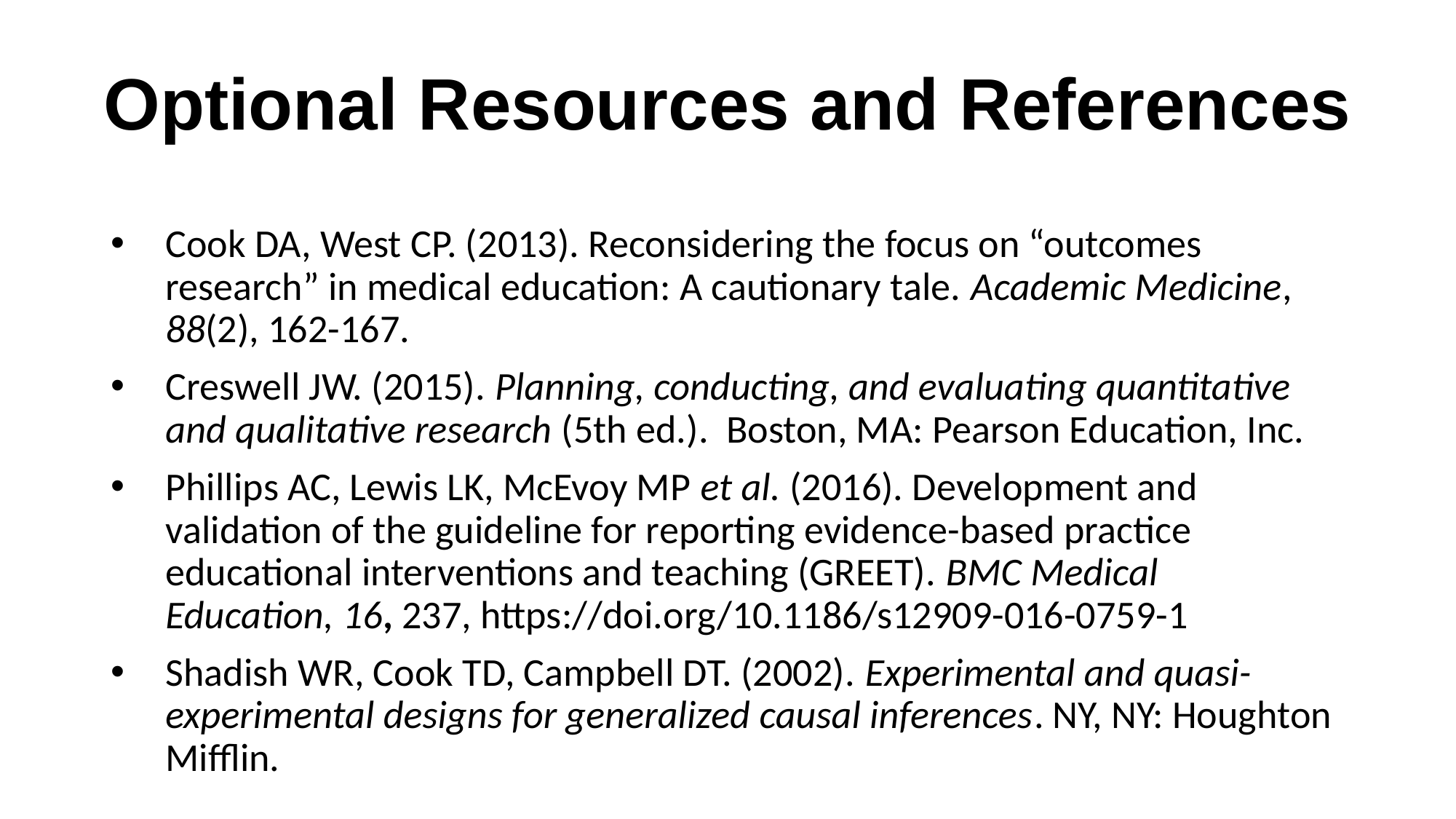

# Optional Resources and References
Cook DA, West CP. (2013). Reconsidering the focus on “outcomes research” in medical education: A cautionary tale. Academic Medicine, 88(2), 162-167.
Creswell JW. (2015). Planning, conducting, and evaluating quantitative and qualitative research (5th ed.). Boston, MA: Pearson Education, Inc.
Phillips AC, Lewis LK, McEvoy MP et al. (2016). Development and validation of the guideline for reporting evidence-based practice educational interventions and teaching (GREET). BMC Medical Education, 16, 237, https://doi.org/10.1186/s12909-016-0759-1
Shadish WR, Cook TD, Campbell DT. (2002). Experimental and quasi-experimental designs for generalized causal inferences. NY, NY: Houghton Mifflin.

## Slide 29
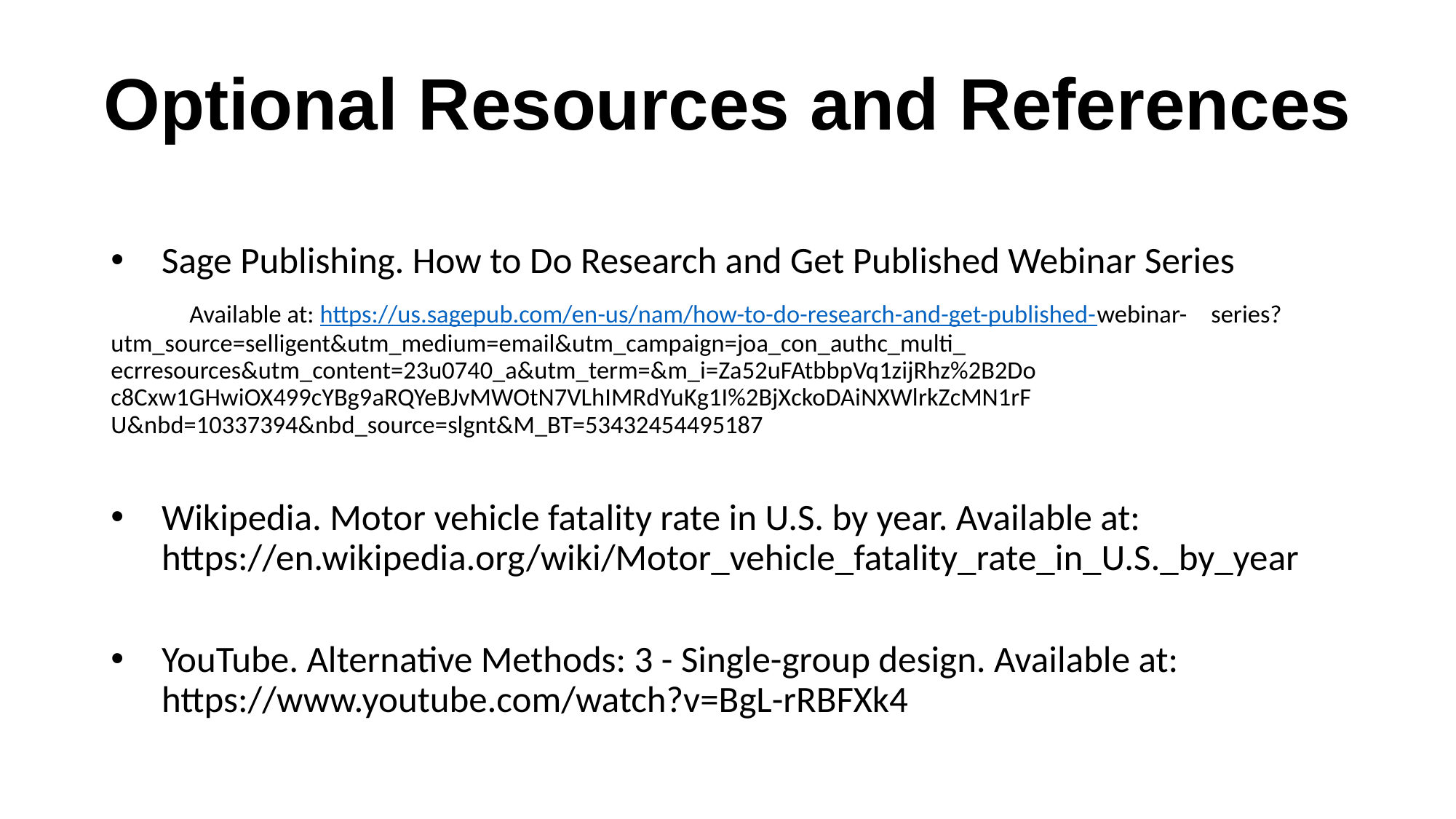

# Optional Resources and References
Sage Publishing. How to Do Research and Get Published Webinar Series
	Available at: https://us.sagepub.com/en-us/nam/how-to-do-research-and-get-published-webinar-	series?utm_source=selligent&utm_medium=email&utm_campaign=joa_con_authc_multi_	ecrresources&utm_content=23u0740_a&utm_term=&m_i=Za52uFAtbbpVq1zijRhz%2B2Do	c8Cxw1GHwiOX499cYBg9aRQYeBJvMWOtN7VLhIMRdYuKg1I%2BjXckoDAiNXWlrkZcMN1rF	U&nbd=10337394&nbd_source=slgnt&M_BT=53432454495187
Wikipedia. Motor vehicle fatality rate in U.S. by year. Available at: https://en.wikipedia.org/wiki/Motor_vehicle_fatality_rate_in_U.S._by_year
YouTube. Alternative Methods: 3 - Single-group design. Available at: https://www.youtube.com/watch?v=BgL-rRBFXk4
