# Supplementary material for: Moving Beyond Simplistic Research Design in Health Professions Education: What a One-Group Pretest-Posttest Design Will Not Prove
Source: MedEdPORTAL. 2025 May 20;21:11527. doi: 10.15766/mep_2374-8265.11527 (PMC12089416; doi:10.15766/mep_2374-8265.11527)
Supplement: Supplementary file 1 — Presentation for Research in HPE.pptxLesson Plan - 60 minutes - In Person.docxLesson Plan - 60 minutes - Virtual.docxLesson Plan - 75 minutes - In Person.docxLesson Plan - 75 minutes - Virtual.docxCase Study and Internal Validity Handout.docxEvaluation Form.docx [file mep_2374-8265.11527-s001.zip › C. Lesson Plan - 60 Minutes - Virtual.docx]

**Appendix C**

**60-Min Lesson Plan for a Live, Virtual Workshop**

**Workshop Title:** Moving Beyond Simplistic Research Design in Health Professions Education:

What a One-group Pretest-Posttest Design Will Not Prove

**Workshop date: Time:** 60 minutes **Facilitators:**

| **Time** | **Minutes** | **Activity** | **Faculty** |
| --- | --- | --- | --- |
|  | 2-3 min | **Slides 1-2:**  #1 – Title slide: Introductions – 2 min  #2 – Acknowledgements, Faculty disclosures and CME – 1 min |  |
|  | 6-7 min | **Slides 3-6**  #3 – Points to Ponder – 2-3 min  #4 – Learning objectives – 1 min  #5 – Definition – 1 min  #6 – Spurious correlation– 1-2 min |  |
|  | 9-12 min | **Slides 7-12**  #7 – Large group question – 2-3 min  #8 – Debrief – 2-3 min  #9 – Graphic – 30 sec (optional)  #10 – Graphic – 30 sec (optional)  #11 – Causation – 2 min  #12 – One group, pre/post designs – 2-3 min with student example |  |
|  | 16-20 min | **Slides 13-15**  #13 – Directions for activity and send to breakout rooms – 2 min  Breakout rooms – 10-12 min for discussion  #14-15 – Debrief case scenario 5-6 min |  |
|  | 5-7 min | **Slides 16-18**   - Designs and examples |  |
|  | 4-6 min | **Slides 19-21**  #19 – Scholarship of teaching – 1 min (optional)  #20 – Alternative scholarly approaches – 2-3 min  #21 – Alternative scholarly approaches – 2-3 min |  |
|  | 4-5 min | **Slides 22-24**  #22 – Other advice – 2 min  #23 – Take aways – 3 min  #24 - Workshop evaluation form (Advance while discussing Take aways) |  |
| **IF TIME** | | **Slides 25-28**  #25 – Closing Advice – 10 secs  #26 – Questions from audience  #27-28 – Optional resources and references |  |
