# Supplementary material for: Moving Beyond Simplistic Research Design in Health Professions Education: What a One-Group Pretest-Posttest Design Will Not Prove
Source: MedEdPORTAL. 2025 May 20;21:11527. doi: 10.15766/mep_2374-8265.11527 (PMC12089416; doi:10.15766/mep_2374-8265.11527)
Supplement: Supplementary file 1 — Presentation for Research in HPE.pptxLesson Plan - 60 minutes - In Person.docxLesson Plan - 60 minutes - Virtual.docxLesson Plan - 75 minutes - In Person.docxLesson Plan - 75 minutes - Virtual.docxCase Study and Internal Validity Handout.docxEvaluation Form.docx [file mep_2374-8265.11527-s001.zip › D. Lesson Plan - 75 Minutes - In Person.docx]

**Appendix D**

**75-Min Lesson Plan for an In Person Workshop**

**Workshop Title:** Moving Beyond Simplistic Research Design in Health Professions Education:

What a One-group Pretest-Posttest Design Will Not Prove

**Workshop date: Time:** 75 minutes **Facilitators:**

| **Time** | **Minutes** | **Activity** | **Faculty** |
| --- | --- | --- | --- |
|  | 3-4 min | **Slides 1-2**  #1 – Title slide: Introductions – 2-3 min  #2 – Acknowledgements, Faculty disclosures and CME – 1 min |  |
|  | 6-7 min | **Slides 3-6**  #3 – Points to Ponder – 2-3 min  #4 – Learning objectives – 1 min  #5 – Definition – 1 min  #6 – Spurious correlation – 1-2 min |  |
|  | 9-12 min | **Slides 7-12**  #7 – Large group question – 2-3 min  #8 – Debrief – 2-3 min  #9 – Graphic – 30 sec (optional)  #10 – Graphic – 30 sec (optional)  #11 – Causation – 2 min  #12 – One group, pre/post designs – 2-3 min with student example |  |
|  | 18-25 min | **Slides 13-15**  #13 – Directions for activity 1-2 min  Discussions at tables – 10-15 min  #14-15 – Debrief case scenario with groups -7-8 min |  |
|  | 8-11 min | **Slides 16-18**  #16 – Ask audience - 2-3 min  #17 – Add one or more groups 3- 4 min  #18 – Add layers – 3-4 min |  |
|  | 4-6 min | **Slides 19-21**  #19 – Scholarship of teaching – 1 min (optional)  #20 – Alternative scholarly approaches – 2-3 min  #21 – Alternative scholarly approaches – 2-3 min |  |
|  | 6-8 min | **Slides 22-25**  #22 – Other advice – 2-3 min  #23 – Take aways – 3-4 min  #25 – Closing advice – 1 min |  |
| **IF TIME** | | **Slides 26-28**  #26 – Questions from audience  #27-28 – Optional resources and references |  |
