# Supplementary material for: Moving Beyond Simplistic Research Design in Health Professions Education: What a One-Group Pretest-Posttest Design Will Not Prove
Source: MedEdPORTAL. 2025 May 20;21:11527. doi: 10.15766/mep_2374-8265.11527 (PMC12089416; doi:10.15766/mep_2374-8265.11527)
Supplement: Supplementary file 1 — Presentation for Research in HPE.pptxLesson Plan - 60 minutes - In Person.docxLesson Plan - 60 minutes - Virtual.docxLesson Plan - 75 minutes - In Person.docxLesson Plan - 75 minutes - Virtual.docxCase Study and Internal Validity Handout.docxEvaluation Form.docx [file mep_2374-8265.11527-s001.zip › F. Case and Internal Validity Handout.docx]

**Appendix F**

**Case Study and Handout for Small Group Activity during Workshop**

**Small Group Activity**

**Directions:** Read the health professions education scenario below before completing the checklist on the next page.

An interprofessional group of educators would like to publish the results of a six-month interprofessional education (IPE) elective. The novel experience (intervention) was designed to allow a group of students to interact with and follow a patient’s family from clinic to hospital to rehabilitation to home.

- All learners in the pilot program (nursing students, medical students, pharmacy students, social work students (n = 50) completed a written pretest on Day 1 (O1) of the IPE experience and an online posttest (O2) were identical and designed to measure “knowledge of interprofessional practice” (outcome variable).
- Students were recruited based upon availability and interest in IPE.
- Coincidentally, medical students completed a free clinic rotation during the same time period. Systems-based practice (including interprofessional roles) was stressed during the rotation.
- Five students could not complete the six-month elective experience due to academic and health reasons.
- The pharmacy students were so motivated after the project started (and prior to the post-test) that they became IPE ambassadors in their own cohort.
- Faculty are excited about publishing the results as scores on the posttest were significantly higher than the pretest.

With your group, complete the checklist on the next page to analyze the scenario and identify threats to internal validity. **Will the team be able to link results to the intervention or are there rival explanations?**

**Threats to Internal Validity Checklist**

**One-Group Pretest-Posttest Design**

| **Threats to Internal Validity** | **Definitions** | **Which threats can you identify?**  (Check all that apply) |
| --- | --- | --- |
| **Attrition/Mortality** | The change in the outcome (*knowledge of interprofessional practice*) was due to participants withdrawing from the study, failing to complete posttest, etc. |  |
| **History** | An event or environmental condition other than the intervention could have caused the change in the outcome variable (*knowledge of interprofessional practice*). |  |
| **Instrumentation** | Observed changes in the outcome variable (*knowledge of interprofessional practice*) were due to inconsistency in the measurement, administration, raters. |  |
| **Maturation** | The change in outcome variable (*knowledge of interprofessional practice*) was due to natural changes that occur over time (due to learning, aging, etc.) and not the intervention. |  |
| **Selection Bias** | Can occur if participants are not randomly selected to participate. This may be a threat if participants volunteer based on interest. |  |
| **Testing** | Observed changes in the outcome variable (*knowledge of interprofessional practice*) were due to previous exposure to a test and not the intervention. |  |
| **Regression to the mean** may also be a threat to internal validity based on participant selection. Those selected with extremely high or low scores will move (regress) to the average (mean) score on subsequent tests. | | |
| Based upon Adams & Lawrence: Research methods, Statistics and Applications. Chap 8. Examining Causal Relationships Among Your Variables. 2015. | | |
