# Supplementary material for: Moving Beyond Simplistic Research Design in Health Professions Education: What a One-Group Pretest-Posttest Design Will Not Prove
Source: MedEdPORTAL. 2025 May 20;21:11527. doi: 10.15766/mep_2374-8265.11527 (PMC12089416; doi:10.15766/mep_2374-8265.11527)
Supplement: Supplementary file 1 — Presentation for Research in HPE.pptxLesson Plan - 60 minutes - In Person.docxLesson Plan - 60 minutes - Virtual.docxLesson Plan - 75 minutes - In Person.docxLesson Plan - 75 minutes - Virtual.docxCase Study and Internal Validity Handout.docxEvaluation Form.docx [file mep_2374-8265.11527-s001.zip › G. Evaluation Form.docx]

**Appendix G**

**Evaluation Form to Distribute at the End of the Workshop**

Moving Beyond Simplistic Research Design in Health Professions Research:

What a One-Group, Pretest-Posttest Design Will Not Prove

**Workshop Evaluation Form**

**Directions:** Please help us to improve this workshop for future audiences by providing your feedback below.

| **As a result of participating in this workshop, I am better able to:** | **Strongly Disagree** | **Disagree** | **Agree** | **Strongly Agree** |
| --- | --- | --- | --- | --- |
| Distinguish between causation versus correlation in health professions education research | ❒ | ❒ | ❒ | ❒ |
| Discuss the concept of internal validity | ❒ | ❒ | ❒ | ❒ |
| Discuss threats to internal validity when using a one group, pre-test/posttest design | ❒ | ❒ | ❒ | ❒ |
| Discuss alternative approaches to evaluate educational innovations | ❒ | ❒ | ❒ | ❒ |
| **Overall, today’s workshop….** | **Strongly Disagree** | **Disagree** | **Agree** | **Strongly Agree** |
| Was well organized | ❒ | ❒ | ❒ | ❒ |
| Was a valuable use of my time | ❒ | ❒ | ❒ | ❒ |
| Gave me ideas I can apply to educational scholarship projects | ❒ | ❒ | ❒ | ❒ |
| Was interactive | ❒ | ❒ | ❒ | ❒ |
| Included knowledgeable faculty facilitators | ❒ | ❒ | ❒ | ❒ |

**What “tip” or “pearl”, if any, did you glean from this workshop?**

**What worked well in this workshop?**

**Please provide at least one recommendation for improving today’s workshop:**
