# Supplementary material for: Moving Beyond Simplistic Research Design in Health Professions Education: What a One-Group Pretest-Posttest Design Will Not Prove
Source: MedEdPORTAL. 2025 May 20;21:11527. doi: 10.15766/mep_2374-8265.11527 (PMC12089416; doi:10.15766/mep_2374-8265.11527)
Supplement: Supplementary file 1 — Presentation for Research in HPE.pptxLesson Plan - 60 minutes - In Person.docxLesson Plan - 60 minutes - Virtual.docxLesson Plan - 75 minutes - In Person.docxLesson Plan - 75 minutes - Virtual.docxCase Study and Internal Validity Handout.docxEvaluation Form.docx [file mep_2374-8265.11527-s001.zip › _Educational Summary Report_11527.pdf]

MedEdPORTAL® The AAMC Journal of Teaching and Learning Resources

Original Publication

Open Access

Moving Beyond Simplistic Research Design in Health Professions Education: What a One-Group Pretest-Posttest Design Will Not Prove

S. Beth Bierer, PhD*, Gary Beck Dallaghan, PhD, Nicole J. Borges, PhD, Sam Brondfield, MD, MAEd, Cha Chi Fung, PhD, Kathryn N. Huggett, PhD, Cayla R. Teal, PhD, Satid Thammasitboon, MD, MHPE, Colleen Y. Colbert, PhD

*Corresponding author: biererb@ccf.org

Abstract

Introduction: Educational research presents unique study design challenges. Novice researchers in health professions education (HPE) frequently misuse the one-group pretest-posttest design, highlighting the need for improved training in research design. This workshop aimed to enhance understanding of research design among novice HPE researchers, specifically addressing the inherent limitations of the one-group pretest-posttest design and offering alternative approaches. **Methods:** Experienced HPE researchers developed this workshop to address common misunderstandings of research design. Leaders from the AAMC Medical Education, Scholarship, Research, and Evaluation section facilitated 60–75-minute workshops conducted at the four 2024 regional meetings hosted by the AAMC Group on Educational Affairs (GEA). Workshop activities included large-group discussion, small-group case-based discussion, and critiques of research designs. Participants discussed internal validity threats and alternative research designs and scholarly approaches. **Results:** Approximately 120 GEA regional meeting registrants attended, with 74 (61%) completing a feedback questionnaire immediately after the workshop. Most respondents reported achieving the workshop's educational objectives, such as being better able to identify internal validity threats associated with the one-group pretest-posttest design (100%) and to discuss alternative approaches to evaluate educational innovations (100%). Additionally, >95% of respondents agreed that the workshop was well organized, interactive, and valuable in providing content they could apply to their educational scholarship. **Discussion:** The workshop successfully clarified misconceptions surrounding the one-group pretest-posttest design while introducing participants to more rigorous research approaches. Facilitator expertise is essential. Future iterations should consider participants' experiences to tailor content further and expand offerings about research methodologies.

Keywords
Educational Scholarship, Research Design, Faculty Development, Publishing/Scholarship, Editor's Choice

Educational Objectives

By the end of this activity, learners will be able to:

1. Distinguish between causation versus correlation in health professions educational research.
2. Discuss the concept of internal validity.
3. Discuss threats to internal validity when using a one-group pretest-posttest design.
4. Discuss alternative approaches to evaluate educational innovations.

Citation:
Bierer SB, Beck Dallaghan G, Borges NJ, et al. Moving beyond simplistic research design in health professions education: what a one-group pretest-posttest design will not prove. *MedEdPORTAL*. 2025;21:11527. https://doi.org/10.15766/mep_2374-8265.11527

Introduction

Within health professions education (HPE) fields, research has historically been predominantly clinical or biomedical. HPE research is a newer field and, although research methods between these areas may overlap, educational research presents unique study design challenges.[1] While research methods courses are commonly taught during undergraduate and graduate education at academic health science centers, these courses generally focus on clinical or biomedical research methods rather than educational (social science) research methods.[2-6] Unless related to coursework within a degree-granting program (e.g., MEd or PhD), educational research methods are not commonly taught within HPE curricula.[7] Because many HPE faculty have migrated from nonsocial-sciences disciplines,[8] their familiarity with educational research methods can vary substantially.[9,10] Consequently, the rigor of HPE research

Copyright © 2025 Bierer et al. This is an open-access publication distributed under the terms of the Creative Commons Attribution license.

may suffer as a result of incomplete training in educational research methods.[11]

This lack of rigor is evident in the continued use of the one-group pretest-posttest design by HPE scholars, despite this design's inherent flaws.[12,13] Educators, eager to publish the results of their interventions, may not realize weaknesses of this design and ultimately its inability to prove that an intervention was efficacious. The major limitation in use of the one-group pretest-posttest research design is threats to internal validity (i.e., history, maturation, testing, instrumentation, etc.),[14] which precludes researchers from claiming causality. For example, residency program faculty who are interested in examining the impact of a new mandatory 1-year quality improvement (QI) curriculum on their residents' knowledge of QI methods will not be able to claim causality based upon changes in residents' QI test scores. This design does not control for external contextual factors, such as formal QI initiatives occurring simultaneously at the departmental or institutional level (history), which may have contributed to changes in resident QI knowledge scores. In this example, maturation also imposes a threat given the time span of the intervention. As Knapp[12] has astutely noted, all one can say when using a one-group pretest-posttest design is that a change has occurred, but not that an intervention caused it.

Reasons why researchers resort to the one-group pretest-posttest research design may include the inability to randomize to multiple groups during normal educational practices, lack of knowledge about educational research design, lack of time (e.g., a rush to publication), as well as the lack of clear guidelines, from some journals, dissuading educators from using this design.[12] While some professional societies and institutions offer courses and programs in HPE research, professional development courses specifically focusing on educational research methods appear to be rare and specialty dependent. Peer-reviewed articles may describe principles of HPE research, but articles cannot substitute for professional development opportunities and coursework.[15,16]

A search of *MedEdPORTAL* using the search term *research design* generated seven publications,[17-23] but none covered educational research designs within their core content. A second search using the terms *research method* and *research methodology* generated three publications.[24-26] Of these, only one touched on issues related to the one-group pretest-posttest design but did not explicitly discourage people from using it.[25] Thus, there is a critical need for faculty development in HPE research methods[27] and training regarding this design. The impact on the field of HPE research is significant. Consumers of HPE research, interested in potentially adopting educational interventions, cannot proceed with any confidence regarding the efficacy of interventions if it is based on a one-group pretest-posttest design.

This workshop was designed specifically for novice HPE researcher audiences who have limited to no knowledge of educational research design. The workshop was created to address gaps in training, especially within clinician educator groups, regarding research design, and specifically the use of the one-group pretest-posttest design.

Methods

Workshop Development

The first author (S. Beth Bierer) and last author (Colleen Y. Colbert) have coached and mentored novice researchers over the past 20 years about the perils of using a one-group pretest-posttest design in educational research. The first author also dealt with recurrent misunderstandings about the rigor of this design while teaching a semester-long research methods course on multiple occasions. To help address this knowledge gap for a broader audience, the first and last authors designed a workshop for interprofessional educators (medical and nursing educators, educational team members, etc.) who were participating in educator development programs at the Cleveland Clinic.

In 2023, both the first and last authors met with leaders of the AAMC Medical Education, Scholarship, Research, and Evaluation (MESRE) section to discuss their experiences with this workshop (90-minute in-person and 60-minute live, virtual workshops at Cleveland Clinic). All MESRE leaders, as experienced research mentors and peer reviewers, agreed about the frequent misuse of the one-group pretest-posttest design and the need to support novice educational researchers who inappropriately use this design. First author Bierer added speaker's notes to the workshop's PowerPoint presentation, and last author Colbert created lesson plans for in-person and virtual workshop delivery. We then met and collectively discussed the workshop's content and activities for in-person delivery at the 2024 regional meetings of the AAMC Group on Educational Affairs (GEA), an organization comprising clinical and basic science faculty, administrative staff, and learners involved in medical education. All MESRE leaders served as workshop facilitators at one or more of the GEA regional meetings where the workshop was offered.

Workshop Delivery

We conducted this workshop on four separate occasions as either 60-minute or 75-minute sessions. For in-person delivery,

Copyright © 2025 Bierer et al. This is an open-access publication distributed under the terms of the Creative Commons Attribution license.

the conference room should have round tables for small-group activities and audiovisual equipment to display a PowerPoint presentation. For live, virtual workshops, we recommend using an online platform (e.g., Zoom, Microsoft Teams) that allows for breakout rooms.

All instructors had expertise with research design in HPE contexts and experience with facilitating small- and large-group discussions. We recommend having two skilled facilitators to provide different perspectives and examples during discussion, though one facilitator is sufficient. The PowerPoint presentation (Appendix A) includes a script for facilitators to help standardize workshop delivery across settings. The outline below summarizes the lesson plan and time line for a 60-minute, in-person workshop (Appendix B). Additional lesson plans exist for a 60-minute virtual workshop (Appendix C) and 75-minute in-person or virtual sessions (Appendices D and E).

Introduction and icebreaker (5-6 minutes): Facilitators introduce themselves and note any disclosures. If feasible, ask participants to introduce themselves. The facilitator uses a nonmedical scenario involving self-driving cars (slide 3, Appendix A) as an icebreaker to encourage participants to reflect upon and briefly discuss how a weak research design could not provide sufficient evidence to inform decision-making (i.e., purchase a self-driving car). A review of workshop educational objectives follows (slide 4, Appendix A).

Define terms (2-3 minutes): The facilitator proceeds to introduce key terms—correlation and causation—and provides an example of a spurious correlation to highlight the importance of having a meaningful relationship between two variables (slides 5-7, Appendix A).

Large-group discussion (9-12 minutes): Once participants have a chance to think about the prompt in a debrief (slide 8, Appendix A), the facilitator leads a large-group discussion of alternative explanations to explain the increase in traffic-related fatalities, other than changing the speed limit. This is intended to be a large-group discussion. However, if there are several participants in the audience, it may be better to do a pair-share first for 2 minutes and then have a large-group discussion of alternative explanations (slide 8, Appendix A). The facilitator may choose to spend more time exploring other explanations using graphics (slides 9 and 10, Appendix A).

Explanation of design (5 minutes): The large-group discussion sets the stage to introduce the three conditions essential to establish causation (slide 11, Appendix A), which is an underlying objective of experimental designs. This explanation builds upon the previous examples of having meaningful relationships between two correlated variables and no alternative/rival explanations for results. The facilitator explains the one-group pretest-posttest design and references how this design will not conclusively prove causation despite its frequent use. The facilitator then provides examples of participants, intervention, and outcomes that align with the case study for the small-group activity (Appendix F).

Small-group activity (16-20 minutes): The facilitator asks participants to form small groups (slide 13, Appendix A) and then critiques an example of a one-group pretest-posttest design using a worksheet with internal validity threats listed (Appendix F). Though the handout and case study provide sufficient guidance for participants to identify internal validity threats without needing direct support from facilitators, we still recommend debriefing with participants about internal validity threats (slides 14 and 15, Appendix A).

Other research designs (5-7 minutes): We observed that workshop participants expressed a desire to consider other experimental or quasi-experimental designs that would have fewer internal validity threats than the one-group pretest-posttest design. To address this need, the facilitator briefly reviews other designs for participants to consider and provides examples (slides 16-18, Appendix A). If time permits, ask participants for examples from their work if they used any of the discussed designs.

Alternative scholarly approaches (6-9 minutes): The facilitator offers other approaches (slides 19-21, Appendix A) so that participants may examine educational interventions/innovations beyond the one-group pretest-posttest design.

Workshop wrap-up (10 minutes): The facilitator summarizes common pitfalls to avoid and then asks participants, "What is one thing you will do differently after participating in this workshop?" The workshop concludes with participants completing an electronic evaluation form, followed by an opportunity to ask the facilitators and other participants questions.

Data Collection and Analysis

We designed a web-based workshop evaluation form (Appendix G) to gather participants' feedback at each GEA meeting by projecting a QR code on a slide at the workshop's completion. The online evaluation form consists of nine Likert-type scale items rated on a 4-point response scale (1 = *strongly disagree*, 2 = *disagree*, 3 = *agree*, and 4 = *strongly agree*). Four

Copyright © 2025 Bierer et al. This is an open-access publication distributed under the terms of the Creative Commons Attribution license.

items aligned with objectives of the workshop, and five items addressed delivery of the workshop and its value to participants. There were three open-ended items on the feedback evaluation. Two items asked participants to provide narrative responses to the following questions: "What tip or pearl, if any, did you glean from this workshop?" and "What worked well in this workshop?" In addition, to identify value to participants and workshop strengths/improvements, participants were asked, "Please provide at least one recommendation for improving today's workshop."

For QI purposes, after each regional workshop, the first author (S. Beth Bierer) sent participants' aggregate feedback and comments to facilitators prior to the next workshop offering. Sharing feedback with facilitators after each workshop provided facilitators with the opportunity to anticipate participants' questions and/or modify workshop timing or content iteratively.

All rating-scale evaluation items were compared using IBM SPSS software version 29.0; results are reported as descriptive statistics. Comments from open-ended evaluation questions were grouped into categories using conventional content analysis.[28] This workshop description met the criteria for a quality improvement project as defined by the Cleveland Clinic Institutional Review Board.

Results

Of the estimated 120 GEA attendees across the four regions, 81 initiated the evaluation form using the QR code provided at the end of each regional workshop. A total of 74 participants completed the evaluation form, and four indicated their region but did not complete the remainder of the evaluation form. Response rates ($N = 74$) across the four regions ranged from 19% to 34%.

Evaluation Related to Educational Objectives

Most of the 74 respondents indicated that the educational objectives of the workshop were achieved (e.g., *strongly agree* or *agree*; Table 1).

The majority of participants indicated that they were better able to distinguish between causation versus correlation after participating in this workshop as well as were better able to discuss the concept of internal validity, threats to internal validity when using a one-group pretest-posttest design, and alternative approaches to evaluate educational innovations (Figure).

Evaluation of Quality and Value

Regarding workshop quality and its value to participants, most respondents indicated that the workshop was well organized, interactive, included knowledgeable faculty facilitators, was a valuable use of participants' time, and provided ideas that could be applied to their educational scholarship projects (Table 2).

Participants' Narrative Feedback

A summary of key narrative responses provided by participants on the feedback evaluation form is presented in Table 3. This table provides representative comments organized by category from the content analysis, including categories of confidence, knowledge, suggestions, format and design, content, facilitators, and requested resources, from participants responding to the three open-ended items on the questionnaire.

Discussion

This workshop offered participants the opportunity to work in both large and small groups to examine the one-group pretest-posttest design, explore limitations, and identify threats to internal validity. Workshop participants included both novice and experienced researchers, and the workshop was well received by both. Most participants reported achievement of each educational objective. For facilitators, the workshop provided an opportunity to focus their teaching on a common (and overused) research design as well as utilize their own research experiences to enhance participants' understanding of the design. The materials included in this publication provide other HPE researchers with a mechanism for addressing this design with novice researchers at their own institutions.

Experience with the one-group pretest-posttest design, as well as alternative designs and approaches, is essential for

Table 1. Participant Evaluation of Workshop Educational Objectives ($N = 74$)

Item	*M (SD)*[a]	*n* (%) Strongly Disagree	Disagree	Agree	Strongly Agree
As a result of participating in this workshop, I am better able to:					
Distinguish between causation versus correlation in health professions education research	3.4 (0.68)	2 (2.7)	2 (2.7)	34 (45.9)	36 (48.6)
Discuss the concept of internal validity	3.5 (0.55)		2 (2.7)	36 (48.6)	36 (48.6)
Discuss threats to internal validity when using a one-group pretest/posttest design	3.6 (0.50)			31 (41.9)	43 (58.1)
Discuss alternative approaches to evaluate educational innovations	3.5 (0.50)			39 (52.7)	35 (47.3)

[a]A 4-point Likert-type scale was used for the evaluation survey (1 = *strongly disagree*, 4 = *strongly agree*).

Copyright © 2025 Bierer et al. This is an open-access publication distributed under the terms of the Creative Commons Attribution license.

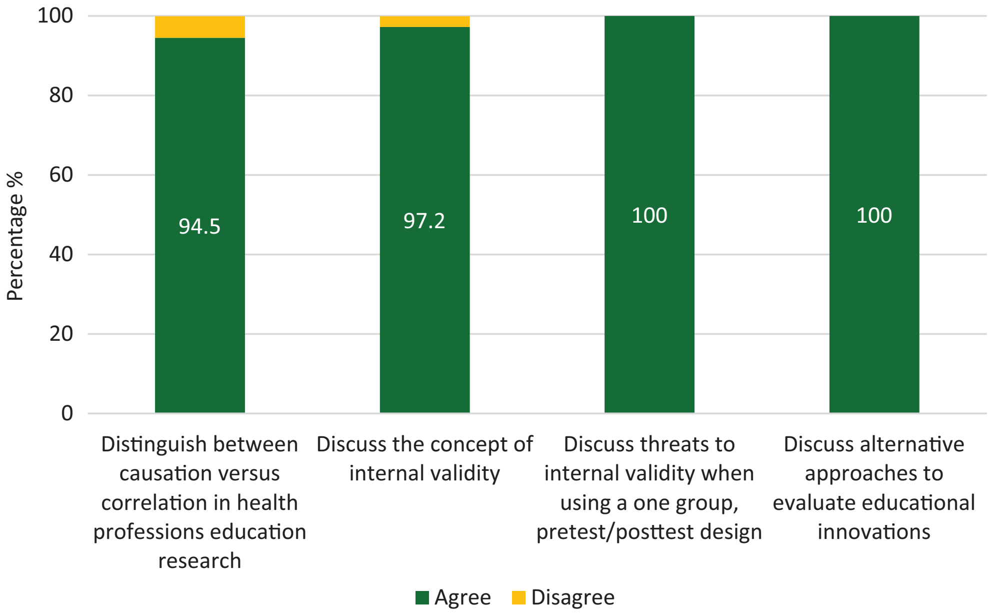

Figure. Feedback on the delivery of the workshop and its value to particpants ($N = 74$) by the percentage that agreed/disagreed with statements based on the question stem "As a result of participating in this wokshop, I am better able to:".

workshop facilitators. We realized early on that, despite our desire to discuss the one-group design in detail, even our less experienced participants rapidly accepted the limitations of the design and wanted alternatives. As a result, the final workshop presentations included more information about alternative research designs, including both additions to the one-group design that could address some threats to validity and the inclusion of additional groups into the design. Though the one-group pretest-posttest design is not useful for assessing the causal effect of an intervention (due to threats to internal validity), it can be appropriately used for other purposes. These purposes were discussed in the workshop and included, for example, using pretest data to identify baseline learner characteristics as a needs assessment and to inform educational QI initiatives.

To broaden participants' familiarity with alternative research approaches, we also discussed participatory action research and qualitative research as well as broader concepts related to the scholarship of teaching and learning. The breadth of questions asked by participants, combined with the wide array of alternatives to the one-group design, necessitated that workshop facilitators had strong research training as well as a variety of research experiences to draw upon. For this reason, team facilitation is particularly appropriate for this workshop; a carefully selected group of facilitators can jointly cover all the topics, as well as more meaningfully answer and discuss participants' questions. Similarly, when more experienced participants, from a variety of backgrounds, asked more nuanced or complex questions, a team of facilitators was better able to consider these questions and address them, without allowing the workshop to digress. While we conducted the workshop in person, those who offered the workshop virtually could, if needed, supplement their own training and experience with facilitators from other institutions. We found it valuable to assign

Table 2. Participant Evaluation of Workshop Quality and Value ($N = 74$)

		n (%)			
**Item**	***M (SD)***[a]	**Strongly Disagree**	**Disagree**	**Agree**	**Strongly Agree**
Overall, today's workshop:					
Was well organized	3.7 (0.47)			23 (31.1)	51 (68.9)
Was a valuable use of my time	3.7 (0.49)		1 (1.4)	20 (27.0)	53 (71.6)
Gave me ideas I can apply to educational scholarship projects	3.6 (0.55)		2 (2.7)	29 (39.2)	43 (58.1)
Was interactive	3.6 (0.52)		1 (1.4)	28 (37.8)	45 (60.8)
Included knowledgeable faculty facilitators	3.8 (0.44)		1 (1.4)	13 (17.6)	60 (81.1)

[a]A 4-point Likert-type scale was used for the evaluation survey (1 = *strongly disagree*, 4 = *strongly agree*).

Copyright © 2025 Bierer et al. This is an open-access publication distributed under the terms of the Creative Commons Attribution license.

Table 3. Representative Comments from Workshop Participants by Category

Question	Category	Quotations
What tip or pearl, if any, did you glean from this workshop?	Confidence	• It was a nice opportunity to check what I am doing in my research design against these potential validity pitfalls<br>• I feel better versed to discuss options when using a single group
	Knowledge	• Learned about two group designs<br>• Really appreciated the framework/checklist and language to use when consulting on projects<br>• Qualitative or mixed methods should always be considered as alternative<br>• Good to challenge a frequently used approach<br>• I learned alternatives to one group pre-test post-test design<br>• The variety of threats to internal validity<br>• Consider other designs than pre/post design<br>• Solomon 4 group design<br>• Multi pretest and posttest may improve conclusions from data. Pivoting to qualitative study design may be better option<br>• Concept of SoTL<br>• Multiple actionable alternative designs for simple pre posttests. Additionally have a more concrete understanding of types of threats to internal validity
	Suggestions	• Handout of the sample/alternative test designs<br>• Spend less time on basics (i.e., correlation vs. causation) and launch immediately into alternative design methods<br>• Walk through an example of curriculum evaluation
What worked well in this workshop?	Format and design	• Good use of case-based instruction<br>• The workshop was organized, the activities worked well to achieve the objectives<br>• Clear materials, good group work<br>• Questions from the audience and specific examples<br>• Love the examples and opportunities to discuss in small group and then debrief with facilitators. Also, it was the right amount of content to cover
	Content	• Reading the study with validity threats and discussing<br>• Helpful slides<br>• Exercise for validity was good
	Facilitators	• Interactivity and expertise of presenters<br>• Faculty were clear and knowledgeable
Please provide at least one recommendation for improving today's workshop	Format	• More activities, perhaps analyses of study designs after being introduced to alternate approaches<br>• More time and more real-life examples of various methodologies
	Content	• More alternatives to one-group pre/post-test design<br>• Include more suggestions for revision of studies to make them more qualitative or mixed methods.<br>• More actual pf [sic] published examples of different kinds of education research
	Requested resources	• A handout to reinforce the alternative research designs

Abbreviation: SoTL, scholarship of teaching and learning.

each workshop facilitator specific aspects of the workshop, in part based upon their unique experiences and skills, and for the group of facilitators to meet and discuss the workshop lesson plan (Appendices B-E).

Some facilitators had 60 minutes for workshop delivery while others had 75 minutes. While the workshop was feasible in either time frame, time management was critical to ensure that discussion of the one-group pretest-posttest design could meaningfully include review of threats to validity as well as allow for rich discussion of alternative research approaches (which came at the end of the workshop). The slightly longer time frame allowed for a workshop that was less rushed, with more robust discussion among participants and facilitators. The design of the workshop, which did not require individual facilitators for the small-group exercise, does accommodate groups of larger size.

Several participants who completed the workshop evaluation said that they appreciated the use of multiple examples and a case for review. However, others suggested that the workshop might benefit from a single research case that could serve as a continuous thread throughout the workshop. For example, the case that we used to have participants consider threats to validity (Appendix F) might also have been used to consider other designs for the case study or alternative approaches. Facilitators agreed that this would be useful to consider when offering the workshop in the future. In addition, it could be helpful to customize the description of the workshop to appeal to the intended audience, such as novice or less experienced educational researchers, who are mostly likely to benefit from the workshop materials and presentation and less likely to ask complex or nuanced questions that take more time to address.

Copyright © 2025 Bierer et al. This is an open-access publication distributed under the terms of the Creative Commons Attribution license.

Success of this workshop is limited by several factors. Facilitator training, skills, and breadth of experience are essential. If the target audience is health professions educators, facilitator experience in educational research is valuable. As noted earlier, use of facilitator teams may help address this need, and team composition should be thoughtfully considered. Additionally, time management is crucial to achieve all workshop objectives and offer participants information that helps them move forward in their own research journeys. The original workshop was modified to include more information about alternative designs and approaches, but doing so requires that facilitators actively manage workshop flow. The lesson plans (Appendices B-E) should help with this. We did not collect demographic data about participants' backgrounds (e.g., faculty rank) or prior experiences with research design on the workshop evaluation. Additional information about participants could be useful in designing future workshops of this type. Anecdotally, more seasoned researchers indicated to us that they attended to observe teaching strategies utilized in this workshop. Finally, we did not receive a 100% response rate on the workshop evaluation form offered to participants immediately after the conclusion of the workshop. We do not know if nonrespondents would have perceptions similar to those of respondents or if using a QR code to access the workshop evaluation adversely affected completion of the workshop evaluation.

Overall, participant feedback from workshop evaluations and our own observations as facilitators suggests that this workshop has value for participants, given the common usage of this design. Participants appreciated the focus on design limitations, as well as alternatives. As such, the workshop has the potential to improve research design among those who might rely on this design. The workshop guides less experienced educational researchers to consider design limitations as well as some starting points for considering more rigorous designs or alternative approaches. Virtual offerings of the workshop allow for expanded access and can aid institutions with few educational research mentors. Future directions should focus on creation of similar workshops, an on-demand lesson, or other readily accessible materials about other commonly used designs for the novice HPE researcher.

Appendices

A. Presentation for Research in HPE.pptx

B. Lesson Plan - 60 minutes - In Person.docx

C. Lesson Plan - 60 minutes - Virtual.docx

D. Lesson Plan - 75 minutes - In Person.docx

E. Lesson Plan - 75 minutes - Virtual.docx

F. Case Study and Internal Validity Handout.docx

G. Evaluation Form.docx

All appendices are peer reviewed as integral parts of the Original Publication.

S. Beth Bierer, PhD: Professor, Department of Medicine, and Director of Assessment and Evaluation, Cleveland Clinic Lerner College of Medicine, Case Western Reserve University School of Medicine; ORCID: https://orcid.org/0000-0002-7952-8822

Gary Beck Dallaghan, PhD: Assistant Dean for Accreditation, Carle Illinois College of Medicine

Nicole J. Borges, PhD: Edward Tulloh Krumm Professor of Medical Education and Chair, Department of Medical Education, Geisel School of Medicine at Dartmouth

Sam Brondfield, MD, MAEd: Associate Professor, Department of Medicine, University of California, San Francisco, School of Medicine

Cha Chi Fung, PhD: Professor, Department of Clinical Medical Education, Vice Chair of Medical Education, and Assistant Dean for Research and Scholarship in Medical Education, Keck School of Medicine of the University of Southern California

Kathryn N. Huggett, PhD: Robert Larner, MD, '42 Professor of Medical Education, Assistant Dean for Medical Education, and Director of the Teaching Academy, Robert Larner, M.D., College of Medicine at the University of Vermont

Cayla R. Teal, PhD: Education Professor, Population Health, and Associate Dean for Assessment and Evaluation, Office of Medical Education, University of Kansas School of Medicine

Satid Thammasitboon, MD, MHPE: Associate Professor, Department of Pediatrics, and Director, Center for Research, Innovation and Scholarship in Health Professions Education, Baylor College of Medicine

Colleen Y. Colbert, PhD: Professor, Department of Medicine, Cleveland Clinic Lerner College of Medicine, Case Western Reserve University School of Medicine, and Director, Office of Educator & Scholar Development, Cleveland Clinic Education Foundation

Acknowledgments
The authors thank Dr. Yichen Zhao, Director of Evaluation and Assessment of the Larner College of Medicine at the University of Vermont, for analyzing data for this submission.

Disclosures
None to report.

Funding/Support
None to report.

Copyright © 2025 Bierer et al. This is an open-access publication distributed under the terms of the Creative Commons Attribution license.

Ethical Approval

The Cleveland Clinic Institutional Review Board reviewed this project.

References

1. Maxfield CM, Thorpe MP, Desser TS, et al. Can the use of deception be justified in medical education research? A point/counterpoint and case study. *Acad Radiol*. 2022;29(7): 1091-1094. https://doi.org/10.1016/j.acra.2021.05.008
2. Ramdani Y, Kurniati Syam N, Karyana Y, Herawati D. Problem-based learning in research method courses: development, application and evaluation. *F1000Res*. 2022;11: 378. https://doi.org/10.12688/f1000research.75985.1
3. Gupta VS, Meier J, Nunez JH, et al. How we did it: implementing a trainee-focused surgical research curriculum and infrastructure. *J Surg Educ*. 2022;79(1):35-39. https://doi.org/10.1016/j.jsurg.2021.07.010
4. Lucas C, Shum SB, Liu M, Bebawy M. Implementing a novel software program to support pharmacy students' reflective practice in scientific research. *Am J Pharm Educ*. 2021;85(10): 1021-1030. https://doi.org/10.5688/ajpe8320
5. Bauer AM, Cerimele JM, Ratzliff A. Development of research methods curriculum for an integrated care fellowship. *Gen Hosp Psychiatry*. 2021;71:55-61. https://doi.org/10.1016/j.genhosppsych.2021.04.007
6. Weeda ER, Weant KA. Development of a pharmacy residency research certificate program. *Hosp Pharm*. 2021;56(2):72-76. https://doi.org/10.1177/0018578719867651
7. Tekian A, Harris I. Preparing health professions education leaders worldwide: a description of masters-level programs. *Med Teach*. 2012;34(1):52-58. https://doi.org/10.3109/0142159X.2011.599895
8. Jensen JL, Bailey EG, Kummer TA, Weber KS. Using backward design in education research: a research methods essay. *J Microbiol Biol Educ*. 2017;18(3):18.3.50. https://doi.org/10.1128/jmbe.v18i3.1367
9. Lai HY, Chen MM, Chen CT, Chang TW, Lee ST, Lee CY. A scoping review of medical education research in neurosurgery. *World Neurosurg*. 2019;126:e1293-e1301. https://doi.org/10.1016/j.wneu.2019.03.080
10. Patel MD, Jordan SG. Medical education research design. *J Am Coll Radiol*. 2022;19(6):693-698. https://doi.org/10.1016/j.jacr.2022.01.019
11. Han H, Youm J, Tucker C, et al. Research methodologies in health professions education publications: breadth and rigor. *Acad Med*. 2022;97(11)(suppl):S54-S62. https://doi.org/10.1097/ACM.0000000000004911
12. Knapp TR. Why is the one-group pretest-posttest design still used? *Clin Nurs Res*. 2016;25(5):467-472. https://doi.org/10.1177/1054773816666280
13. Spurlock DR Jr. The single-group, pre- and posttest design in nursing education research: it's time to move on. *J Nurs Educ*. 2018;57(2):69-71. https://doi.org/10.3928/01484834-20180123-02
14. Campbell DT, Stanley JC. Three pre-experimental designs. In: Campbell DT, Stanley JC. *Experimental and Quasi-Experimental Designs for Research*. Houghton Mifflin Company; 1963:6-12.
15. King E, Norman EJ, Mossop LH, et al. Qualitative research in veterinary medical education: part 1—principles of qualitative design. *J Vet Med Educ*. 2021;48(5):511-518. https://doi.org/10.3138/jvme-2019-0101-p1
16. McOwen KS, Varpio L, Konopasky AW. How to … use theory as method in HPE research. *Clin Teach*. 2023;20(6):e13615. https://doi.org/10.1111/tct.13615
17. Arps K, Schulman D, Tigges S. Confounding and interaction: an interactive self-study quiz. *MedEdPORTAL*. 2014;10:9912. https://doi.org/10.15766/mep_2374-8265.9912
18. Palanisamy D, Xiong W. An interactive approach to teaching the clinical applications of autonomy and justice in the context of discharge decision-making. *MedEdPORTAL*. 2020;16:10992. https://doi.org/10.15766/mep_2374-8265.10992
19. Fenton K, Kim J, Abramson E, Waggoner-Fountain L, Naifeh M, Li ST. Mentoring resident scholarly activity: a toolkit and guide for program directors, research directors and faculty mentors. *MedEdPORTAL*. 2015;11:10103. https://doi.org/10.15766/mep_2374-8265.10103
20. Bower D, Webb T, Larson G, et al. Patient-centered care workshop: providing quality health care to a diverse population. *MedEdPORTAL*. 2007;3:579. https://doi.org/10.15766/mep_2374-8265.579
21. Green C, Li ST, Jirasevijinda T, Bostwick S, Howell J, Abramson E. Research mentorship for medical trainees across the continuum: a faculty toolkit. *MedEdPORTAL*. 2015;11:10132. https://doi.org/10.15766/mep_2374-8265.10132
22. Coplen A, Klausner C, Bleed M, Johnson L. Dentistry case: down syndrome (Thomas) (Out of Print). *MedEdPORTAL*. 2009;5:5107. https://doi.org/10.15766/mep_2374-8265.5107
23. Wessel C. Responsible literature searching for research: a self-paced interactive educational program (Out of Print). *MedEdPORTAL*. 2006;2:162. https://doi.org/10.15766/mep_2374-8265.162
24. Childs E, Demers LB. Qualitative coding boot camp: an intensive training and overview for clinicians, educators, and administrators. *MedEdPORTAL*. 2018;14:10769. https://doi.org/10.15766/mep_2374-8265.10769
25. Lebeau R. Guiding educational research projects: activity-based workshops on writing a literature review and developing research questions. *MedEdPORTAL*. 2015;11:10143. https://doi.org/10.15766/mep_2374-8265.10143

Copyright © 2025 Bierer et al. This is an open-access publication distributed under the terms of the Creative Commons Attribution license.

26. Blanchard R, Scott J. Connecting mixed methods as an education research strategy. *MedEdPORTAL*. 2014;10:9670. https://doi.org/10.15766/mep_2374-8265.9670

27. Behar-Horenstein LS, Beck DE, Su Y. Perceptions of pharmacy faculty need for development in educational research. *Curr Pharm Teach Learn*. 2018;10(1):34-40. https://doi.org/10.1016/j.cptl.2017.09.019

28. Hsieh HF, Shannon SE. Three approaches to qualitative content analysis. *Qual Health Res*. 2005;15(9):1277-1288. https://doi.org/10.1177/1049732305276687

Received: December 12, 2024
Accepted: April 4, 2025
Published: May 20, 2025

Copyright © 2025 Bierer et al. This is an open-access publication distributed under the terms of the Creative Commons Attribution license.
